# Assessing limits of sustainable seed harvest in wild plant populations

**DOI:** 10.1111/cobi.70075

**Published:** 2025-05-31

**Authors:** Anna Bucharova, Oliver Bossdorf, J. F. Scheepens, Roberto Salguero‐Gómez

**Affiliations:** ^1^ Department of Biology Philipps‐University Marburg Marburg Germany; ^2^ Institute of Evolution and Ecology University of Tübingen Tübingen Germany; ^3^ Faculty of Biological Sciences Goethe University Frankfurt Frankfurt am Main Germany; ^4^ Department of Biology University of Oxford Oxford UK

**Keywords:** COMPADRE, ecological restoration, matrix population models, plant demography, plant population, seed harvesting, COMPADRE, cosecha de semillas, demografía botánica, modelos de matriz poblacional, población de plantas, restauración ecológica, **关键词**: 种子采收, 种群矩阵模型, 生态恢复, 植物种群统计, 植物种群, COMPADRE数据库

## Abstract

Seed harvesting from wild plant populations is key for ecological restoration, but it may threaten the persistence of the source populations. Consequently, several countries have set guidelines limiting the proportions of harvestable seeds. However, these guidelines are inconsistent and lack a solid empirical basis. We used matrix population models based on 280 wild plant species, stored in he COMPADRE Plant Matrix Database, to model the demographic consequences of seed harvesting. Current guidelines do not protect populations of annuals and short‐lived perennials because maximal allowed harvest drew all annual species included in our study to extinction. In contrast, current guidelines are overly restrictive for long‐lived plants because these plants could tolerate even higher seed removal than currently allowed. The maximum possible fraction of seed production that can be harvested without compromising the long‐term persistence of populations was strongly related to generation time of the target species. When harvesting seeds every year, the fraction of seeds that was safe to harvest (safe seed fraction) ranged from 80% in long‐lived species to 2% in most annuals. Less frequent seed harvesting substantially increased the safe seed fraction. In the most vulnerable annual species, it was safe to harvest 5%, 10%, or 30% of a population's seed production when harvesting every 2, 5, or 10 years, respectively. Our results provide a quantitative basis for seed harvesting legislation, based on the generation times of species and harvesting regime.

## Abstract (es)

Evaluación de los límites de la cosecha sustentable de semillas en poblaciones de plantas silvestres

**Resumen:**

La cosecha de semillas de poblaciones de plantas silvestres es clave para la restauración ecológica, pero puede amenazar la persistencia de las poblaciones de origen. Por ello, varios países han establecido directrices que limitan las proporciones de semillas cosechables. Sin embargo, estas directrices son incoherentes y carecen de una base empírica sólida. Usamos modelos de matriz poblacional basados en 280 especies de plantas silvestres, almacenadas en la base matriz de datos de plantas COMPADRE, para modelar las consecuencias demográficas de la recolección de semillas. Las directrices actuales no protegen a las poblaciones de plantas anuales y perennes de vida corta porque la cosecha máxima permitida llevó a la extinción a todas las especies anuales incluidas en nuestro estudio. Al contrario, las directrices actuales son excesivamente restrictivas para las plantas longevas porque estas plantas podrían tolerar una extracción de semillas aún mayor que la permitida actualmente. La fracción máxima posible de producción de semillas – lo que puede recolectarse sin comprometer la persistencia a largo plazo de las poblaciones – se relacionó fuertemente con el tiempo de generación de la especie objetivo. Cuando se cosechan semillas todos los años, la fracción de semillas que se puede recolectar sin peligro (fracción segura de semillas) oscila entre el 80% en las especies longevas y el 2% en la mayoría de las anuales. La menor frecuencia de cosecha de semillas aumentó sustancialmente la fracción segura de semillas. En las especies anuales más vulnerables, era seguro cosechar el 5%, el 10% o el 30% de la producción de semillas de una población cuando se cosechaba cada dos, cinco o diez años, respectivamente. Nuestros resultados proporcionan una base cuantitativa para la legislación sobre la cosecha de semillas, basada en los tiempos de generación de las especies y el régimen de cosecha.

## 评估野生植物种群可持续种子采收的限度

**【摘要】** 从野生植物种群中采收种子是生态恢复的关键, 但这可能会威胁源种群的续存。因此, 一些国家制定了限制可采收种子比例的指南。然而, 这些指南并不一致, 且缺乏坚实的经验基础。本研究利用COMPADRE植物矩阵数据库中存储的280种野生植物的种群矩阵模型, 模拟了种子采收对种群动态的影响。我们发现, 现有指南并不能保护一年生植物和寿命较短的多年生植物种群, 因为所允许的最大采收量会导致我们研究中所有的一年生植物物种灭绝。相反, 现有指南对寿命较长的植物限制过多, 因为这些植物可以承受比目前允许的更高的采收量。种子产量的最大可用部分, 即在不影响种群长期续存的情况下可以采收的种子量, 与目标物种的世代时长密切相关。在每年都采收种子的情况下, 可安全采收的种子量(安全采种量)从长寿物种中的80%到大多数一年生植物中的2%不等。减少采收频率可大幅提高安全采种量。在最濒危的一年生植物中, 每两年、五年或十年采收一次种子, 分别可安全采收种群种子产量的5%、10%和30%。我们的研究结果为基于物种世代时长和种子采收方式来制定种子采收的法规提供了定量依据。**【翻译:胡怡思;审校:聂永刚】**

## INTRODUCTION

The restoration of degraded ecosystems is a major goal of global nature conservation (IPBES, 2018). In the middle of the United Nations Decade on Ecosystem Restoration (https://www.decadeonrestoration.org/), a key goal is to reverse the destruction and degradation of billions of hectares of ecosystems. However, ecological restoration at such scales requires high volumes of plant seeds for the reestablishment of native vegetation (Hölzel et al., 2012). Although there is a growing industry for the production of wild plant seeds in specialized seed orchards (Conrady et al., 2023; Nevill et al., 2016), it is still common for seeds to be harvested on a large scale from wild populations and used directly for restoration seeding (Broadhurst et al., 2015; Jones, 2019; Stevenson, 2016; White et al., 2018).

With increasing demands for wild plant seeds, there is a growing risk of driving source populations to local extinction (Meissen et al., 2015; Menges et al., 2004). Moreover, donor populations are often remnants of habitats with high conservation value (Meissen et al., 2015; Pedrini et al., 2020). To prevent substantial negative effects on the long‐term viability of these populations, some regions, in particular the United States (Plant Conservation Alliance, 2015), Australia (Yenson et al., 2021), and Europe (ENSCONET, 2009; Prasse et al., 2010), have begun to set limits for the maximum fraction of seeds that can be harvested annually from wild populations (hereafter safe seed fraction). Still, the safe seed fraction guidelines are currently inconsistent across countries, for example, 20% harvest is allowed in the United States (Plant Conservation Alliance, 2015) and 10% in Australia (Yenson et al., 2021), but only 2–10% in Germany, depending on plant growth type (Prasse et al., 2010). When seeds are harvested less often than every year, some guidelines permit higher safe seed fractions (Prasse et al., 2010). In general, however, these guidelines are mostly based on expert opinion and lack a solid quantitative basis.

Experimental tests of the effects of seed harvesting on wild populations are so far rare, and the existing studies have usually focused on individual species or specific ecosystems (Meissen et al., 2015, 2017; Peres et al., 2003). More effective seed harvesting rules would require data from multiple species and ecosystems, but collecting such data is very labour intensive and costly. As an alternative to collecting new data, Menges et al. (2004) used published plant matrix population models to link seed harvesting to the probabilities of population extinction for 22 perennial species. While this study is widely used to underpin seed collection guidelines for *ex situ* conservation of rare species (e.g., Center for Plant Conservation., 2019; Yenson et al., 2021), the species set has been largely limited to herbaceous perennials of temperate and subtropical North America. For a solid, more global‐scale quantitative prediction of the effects of seed harvesting on wild populations, data from many more species across different life histories and ecosystems are needed.

We employed a modeling approach and simulated seed harvesting for 280 plant species, from annuals to long‐lived trees and from many habitats around the globe, with matrix population models stored in the COMPADRE Plant Matrix Database (COMPADRE Plant Matrix Database, 2019) (Appendix S1; Salguero‐Gómez et al., 2015). Specifically, we tested the efficacy of current guidelines at safeguarding long‐term population persistence; identified traits associated with species vulnerability to seed harvesting; and used the trait that best determines species vulnerability to seed harvesting (i.e., generation time) to predict the safe seed fraction and to formulate a quantitative basis for seed harvesting guidelines in wild plant populations worldwide.

## METHODS

We used data stored in the COMPADRE Plant Matrix Database (COMPADRE Plant Matrix Database., 2019) and selected matrix population models for 280 species (Appendix S1). Because the ultimate goal of this study was to simulate seed harvesting, we selected field‐based models for angiosperms with clearly defined sexual reproduction (see Appendix S2.1 for details). For the majority of studies in COMPADRE, matrix population models are available for several annual transitions and populations. For all calculations, except the stochastic simulations (see below), we used a single matrix population model per species, averaged across all years and populations available for that species. Below, we briefly outline our methods. A more detailed description is available in Appendix S2.

### Effectiveness of current guidelines

To test how well current guidelines safeguard long‐term population persistence, we used matrix population models to calculate 30‐year projections of population sizes. We simulated seed harvesting as a reduction of the sexually produced new recruits. We generally modelled the most extreme scenario: the highest permitted seed harvesting fraction every year. To allow comparison across species, we expressed the effects of seed harvesting as relative population sizes, where, for example, 0.8 represents a 20% reduction and 0.3 a 70% reduction of population size over 30 years relative to the population sizes that would be reached without seed harvesting (Appendix S2.3). Because the effects of seed harvesting were independent of the biogeographic origins of the examined species (Appendix S2.4), we generally used all species in our data set to test the guidelines of specific countries. Because in the German guidelines the recommended safe seed fractions are growth‐type specific (Prasse et al., 2010), we present the results separately for different growth types.

### Traits that predict vulnerability to seed harvesting

To identify a better predictor of safe seed fraction than growth type, we examined whether and which life‐history traits were better predictors of seed harvesting impacts (Table 1). To enable practitioners to apply our findings, we restricted our analyses to 5 key life‐history traits readily available from public databases (COMPADRE Plant Matrix Database, 2019; Kattge et al., 2011; Salguero‐Gómez et al., 2015): generation time (the number of years necessary for the individuals of a population to be fully replaced by new ones), mean age at sexual maturity, degree of iteroparity (frequency of reproduction), clonality, and seed bank persistence (Figure 2; Appendix S2.5). The first 3 traits are associated with the main axes of variation in plant life history worldwide (Salguero‐Gómez et al., 2016). Generation time and mean age at sexual maturity are associated with the fast–slow gradient, the main dimension of plant life‐history variation, which represents fast‐growing, short‐lived plant species at one end and slow‐growing, long‐lived species at the other (Salguero‐Gómez et al., 2016). The degree of iteroparity reflects reproductive strategy, the second main dimension of life‐history variation. We further included clonality and seed bank persistence because these traits buffer against seed harvesting impacts in some species (Adams et al., 2005; Meissen et al., 2017). We then related these traits as explanatory variables to the vulnerability of our 280 species to seed harvesting as response variable. We defined vulnerability as the slope of the relative decrease in population size along increasing seed harvesting fraction (Appendices S2.3‐2.6).

### Improved seed harvesting guidelines

To provide a quantitative basis for improving seed harvesting guidelines, we used generation time, the best predictor of species vulnerability to seed harvesting, to estimate the safe seed fractions across species (Appendix S2.7). We defined the safe seed fractions as the proportions of seed production where annual removal caused a <50% decrease in population sizes over 30 years of continuous seed harvesting relative to the same populations without seed harvesting. A 50% decrease over 30 years corresponded to an annual decrease of about 2%. This threshold ensured a >95% probability of population viability under environmental stochasticity in all analyzed species but one (Appendix S2.8).

### Effects of environmental stochasticity

To determine how environmental stochasticity affected our predictions for seed harvesting based on mean matrix population models, we simulated the effects of environmental stochasticity on population dynamics (Appendix S2.8). This was possible in 108 species for which we had at least 3 spatial or temporal replicate matrix population models (so‐called individual models). We simulated environmental stochasticity projecting population vector by randomly drawing individual matrix population models at each step, replicated 1000 times to obtain probability distributions of seed harvesting impacts. To determine how robust our estimates were to environmental stochasticity, we compared the safe seed fractions based on the mean matrix models with the respective median safe seed fractions based on the stochastic simulations (Appendix S2.8). We also used stochastic simulations to test whether the thresholds of 50% population declines (see above) effectively prevented populations from extinction (Appendix S2.8).

## RESULTS

### Effectivity of current guidelines

**FIGURE 1:**
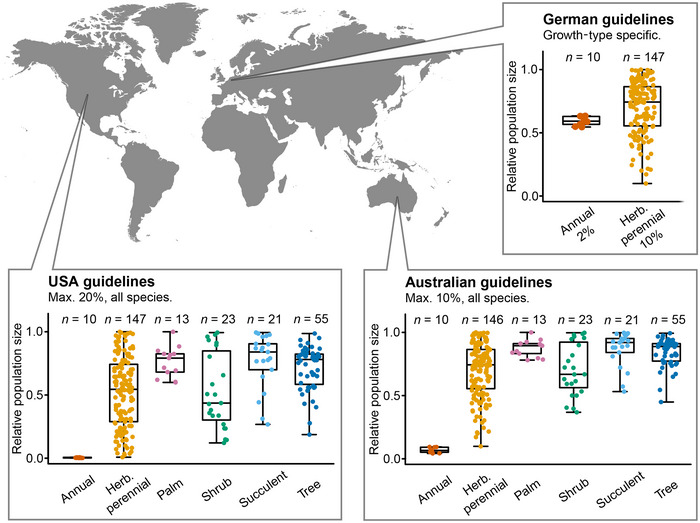
Predicted effects of 30 years of continuous seed harvesting on the relative population sizes of plant species worldwide based on the current guidelines of countries where legislation for seed harvesting exists: United States, Germany, and Australia (points, individual species; Herb., herbaceous; *n*, number of species; whiskers, data range; data from simulation of seed harvesting with matrix population models parameterized with data from natural populations).

Seed harvesting according to the current safe seed fraction guidelines resulted in rather variable relative population sizes among species (Figure 1). For instance, the current U.S. guidelines (20% seed harvesting) were predicted to protect long‐lived palms, with relative population sizes of 0.6–1.0 after 30 years, but to drive all 10 annual plants in our database to extinction (Figure 1). With the more restrictive German guidelines (2% seed harvesting), annual plants were projected to persist, with relative population sizes of 0.54–0.63 after 30 years. For all other plant growth types, the effects of seed harvesting on relative population sizes were highly variable. For example, with the 20% seed harvesting currently allowed in the United States, the predicted relative population sizes of herbaceous perennials ranged from 0 (local extinction) to 1 (no effect) after 30 years, whereas that of shrubs ranged from 0.12 to 0.99, of succulents from 0.27 to 0.99, and of trees from 0.18 to 0.99 (Figure 1).

### Traits that predict vulnerability to seed harvesting

**FIGURE 2:**
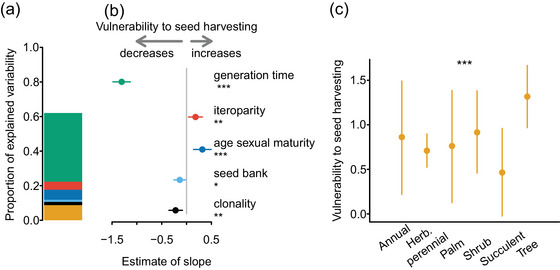
Associations of plant life histories and growth forms with variation in seed harvesting vulnerability across 280 plant species as calculated from matrix population models parameterized with data from natural populations: (a) proportion of variability explained by different life‐history traits and (b) their estimated effect sizes and (c) fitted values of vulnerability for different growth types (error bars, 95% credible intervals; Herb., herbaceous; *p < 0.05; **p < 0.01; ***p < 0.001). Because both vulnerability to seed harvesting and all explanatory variables were standardized prior the analysis, the slope estimates are in arbitrary units. See Appendix S2.6 for detailed model results.

Generation time was the strongest predictor of population vulnerability to seed harvesting. This life‐history trait alone explained 52.3% of the variation in harvesting vulnerability; vulnerability to seed harvesting decreased as generation time increased (Figure 2b). The 4 other examined life‐history traits were also significantly related to seed harvesting vulnerability (Figure 2b). Species that reproduce more frequently or postpone their first reproductive event are more vulnerable to seed harvesting, whereas species with clonal reproduction or persistent seed banks were less vulnerable. However, compared with generation time, the predictive power of the other 4 traits is low (Figure 2a; Appendix S2.6). Population vulnerability also differed significantly among plant growth types, but with minor effects (Figure 2c; Appendix S2.6). All 5 life‐history traits and plant growth type together explained 62.3% of the among‐species variability in vulnerability to seed harvesting.

**TABLE 1:** Definitions of the life‐history traits.

Trait	Description
Generation time	Number of years necessary for the individuals of a population to be fully replaced by new ones
Iteroparity	Frequency of reproduction throughout the lifespan of an individual, with high or low Demetrius’ entropy (*S*) values for highly iteroparous or semelparous populations
Age at sexual maturity	Average number of years after which individuals in a population become sexually reproductive
Seed bank	Mean life expectancy of seeds in the soil seed bank
Clonality	Per‐capita clonal contributions, weighted by the relative frequency of individuals in each stage along the life cycle of the species

### Improved harvesting guidelines

**FIGURE 3:**
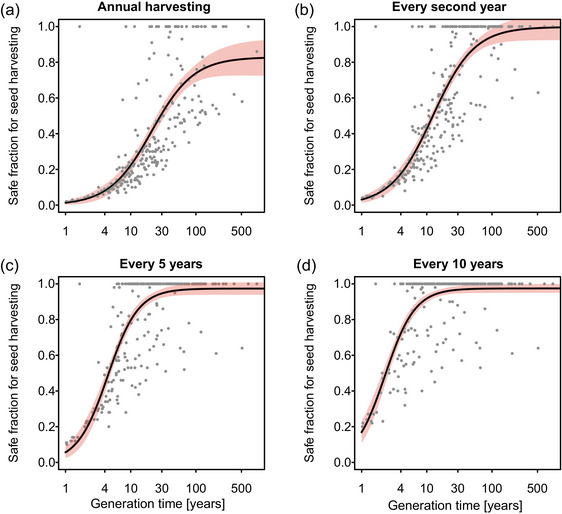
Relationships between the generation times of 280 plant species and the maximum proportion of annual seed production of a population that can be harvested without reducing the relative population size to below 50% in 30 years (safe seed fraction) estimated at different harvesting frequencies (shading, 95% credible interval).

For annual harvesting, the predicted safe seed fractions ranged from close to 0% to 100%. The averages were 2.3% (95% CI 0.5–4.1) for annual and biennial plants, 10.1% (6.8–14.2) for species with a 5‐year generation time, and 40.1% (36.4–43.7) for species with generation times of 20 years (Figure 3a). With harvesting only every 2 years, the safe seed fraction for annuals and biennials was predicted to increase from 2.3% to 5.3% (2.7–7.9). With a 5‐ or 10‐year harvesting interval, the safe seed fraction for annuals and biennials was predicted to increase to 11.3% (6.5–16.0) and 30.3% (23.8–36.8), respectively (Figure 3b–d). For plant species with generation times of 3 or more years, a 5‐year harvesting cycle resulted in a predicted average safe seed fraction of >30% (Figure 3c). In spite of this critical dependence of the safe seed fraction on generation time, there was still substantial residual variation among species with the same generation times.

### Effects of environmental stochasticity

Environmental stochasticity did not substantially affect our estimates of population vulnerability and safe seed fractions. In models that included environmental stochasticity, the median of safe seed fractions was on average 1.8% larger than the median based on the mean models for each species, and they were very closely correlated (Appendix S2.8). The threshold of 50% population decline, which we used as a basis for the new guidelines, ensured >95% probability of population survival under environmental stochasticity in all analyzed species but one (Appendix S2.8).

## DISCUSSION

Seed harvesting in wild populations is indispensable for *ex situ* conservation and ecosystem restoration, but overharvesting can threaten source populations (Pedrini et al., 2020). Consequently, some countries have introduced limits that restrict wild seed harvesting (Plant Conservation Alliance, 2015; Prasse et al., 2010; Yenson et al., 2021). Here, using data from wild populations of 280 plant species from 5 continents, we found that the current seed harvesting guidelines were often ineffective. Existing guidelines did not protect populations of annuals and short‐lived perennials and were overly restrictive for long‐lived plants. Based on generation time, the trait that best predicted seed harvesting vulnerability, we estimated that safe seed fraction varies from 2% in annual and biennial plants to 80–100% in long‐lived plants when seeds are harvested annually. A lower frequency of harvesting allows for higher safe seed fractions. The safe seed fractions suggested here can serve as an improved quantitative basis for seed harvesting regulations globally.

When wild seed harvesting followed the existing safe seed fraction guidelines, the effects on population sizes varied from no effect to extinction, depending on the species. For example, annual harvesting of 20% of the annual seed production, as currently recommended in the United States (Plant Conservation Alliance, 2015), would have a small effect on palms, trees, or some herbaceous perennials, but it would drive all annual plants to extinction within only a few decades. In reality, extinction would be less common because we modelled an extreme scenario when seeds are harvested every growing season for 30 consecutive years from the same population, which is possible but uncommon (Pedrini et al., 2020). Nevertheless, the high variability in model outcomes highlights that effective safe seed fraction guidelines cannot be one size fits all—as currently implemented in many regions (ENSCONET, 2009; Plant Conservation Alliance, 2015; Yenson et al., 2021).

The current German safe seed fractions guidelines are plant‐growth‐type specific (Prasse et al., 2010). For annual plant species, the safe seed fraction is 2% when harvesting annually, which in our model did not cause unacceptable population declines (Figure 1). For herbaceous perennials, the safe seed fraction in Germany is 10% for annual harvest, yet this threshold leads to a wide range of relative population sizes, from substantial population declines to no effects. The variability within the herbaceous perennials is even stronger when following the U.S. guidelines (20% of annual seed production). Plant growth type alone is thus a poor predictor of species vulnerability to seed harvesting, mainly due to the high variability in other relevant life‐history traits within growth types.

Over 60% of the vulnerability to seed harvesting is predicted by life‐history traits. Generation time had the highest predictive value in our analyses and predicted the seed harvesting vulnerability by more than 50% on its own. Population growth rates in long‐lived species are generally rather insensitive to changes in fecundity (Franco & Silvertown, 2004; Janovský & Herben, 2020). Indeed, Menges et al. (2004) showed that long‐lived plants are relatively insensitive to seed harvesting. Other life‐history traits we examined had much smaller predictive power for seed harvesting impacts. For instance, species with higher iteroparity (i.e., reproducing more than once during their life cycle) and species that are sexually mature later in life were more vulnerable to seed harvesting, whereas clonal species and species with permanent soil seed banks were less vulnerable. The buffering effect of soil seed banks against the effects of seed harvesting is well supported by the literature (Adams et al., 2005). However, the relatively small effect of clonality on the impacts of seed harvesting was surprising because clonality provides an alternative reproduction independent of seed production and has been experimentally identified as a major predictor of vulnerability to seed harvesting in grassland plants (Meissen et al., 2017). This discrepancy is likely because many matrix population models calculate generation times of individuals originated from seeds (i.e., genets). Clonal reproduction thus leads to longer generation times of the genets (de Witte & Stöcklin, 2010; Janovský & Herben, 2020) and explains little additional variability in vulnerability to seed harvesting above what is already explained by generation time as the more universal predictor.

To provide a universal quantitative basis for seed harvesting guidelines, we estimated the safe seed fraction as a function of generation time, the best predictor of vulnerability to seed harvesting (Figure 3). The lowest safe seed fractions were in annuals and biennials, 2.3% for annual harvest, which is close to the current German guidelines of 2% (Prasse et al., 2010). The safe seed fraction continuously increased with increasing generation time but remained below 10% for plants with generation times of 5 years and less. Adhering to such low seed safe fractions is possible only when collecting seeds manually, yet this is very labor intensive. In cases where seeds are harvested using combine harvesters, such as in grasslands, 30% of ripe seeds are typically removed (Scotton & Ševčíková, 2017). Such a high proportion is safe for annual harvesting only in species with generation times above 15 years. Grasslands are dominated by annuals and herbaceous perennials, of which 60% in our data set have generation times below 15 years. Annual seed removal with combine harvesters thus threatens a substantial proportion of grassland species, especially nonclonal forbs and annuals (Meissen et al., 2017).

A solution to this problem is to harvest less frequently. Harvesting seeds less often is already suggested as a precautionary principle in some guidelines (e.g., ENSCONET, 2009; Pedrini et al., 2020), although mostly without a clear specification of safe seed fractions and harvesting frequencies. Less frequent harvesting is relevant especially for species with short generation times, where the safe seed fraction is the lowest. In annuals and biennials, the safe seed fraction increased from 2.3% for annual harvesting to 5% when harvesting every second year, to 11% every 5 years, and to 30% every 10 years. Harvesting at 10‐year intervals allows for the collection of 30% of the seeds produced, even in the most vulnerable species. Seed harvesting with a combine harvester, which typically removes the 30% of the ripe seeds, should thus be sustainable even in drylands with a high proportion of annual plants if it is done at sufficiently long intervals.

Seed harvesting is less problematic in species with long generation times. In species with generation times above 20 years (most trees and palms, many shrubs, and some herbaceous perennials [Salguero‐Gómez et al., 2016]), the safe seed fractions were above 40% when harvesting every year and above 80% when harvesting every second year or less frequently. Previous empirical and modeling studies also reported that long‐lived species are rather insensitive to seed harvesting (Meissen et al., 2015, 2017; Menges et al., 2004), although too frequent and too intense harvesting can deplete populations of seedlings (Peres et al., 2003). Even in long‐lived species, it might thus be beneficial to omit seed harvesting in some years to give populations opportunities for juvenile recruitment.

Our results demonstrate the demographic impact of seed harvesting and how it depends on plant life histories. Yet, we could have overestimated harvesting impacts for 3 reasons. First, our analyses are based on matrix population models of species averaged across years and sites, but temporal or spatial variation in demographic rates could buffer some impacts of seed harvesting (Villellas et al., 2015). Indeed, incorporating environmental and demographic stochasticity into our models in a subset of species resulted in safe seed fractions that were on average 1.8% larger, confirming that matrix averaging may cause overestimation, but the effect was small. Second, our approach assumes plant populations to be seed‐limited. However, longer‐lived plants are often limited by safe sites rather than seeds, whereas seed limitation is more common in short‐lived species (Turnbull et al., 2000). It is thus likely that in longer‐lived species, the effects of seed harvesting are even less severe than our findings suggest, but for annuals and short‐lived forbs—the most vulnerable to seed harvesting—our results are more likely to be accurate. A specific case of safe‐site‐limited habitats is European seminatural meadows that are mown annually, and the cut biomass, including a large proportion of seed, is used as fodder for domestic animals. Species growing in this ecosystem are likely adapted to regular seed removal and are thus less vulnerable to seed harvesting than predicted by our models. Third, our models did not incorporate maximal carrying capacities because this information is rarely available for matrix population models. In populations with high population growth rates and close to carrying capacity of the environment, matrix models still predict population growth, even though the populations already reached maximal space occupancy. In such cases, seed harvesting might have a much smaller effect than predicted.

Seed harvesting in wild populations should be generally accompanied by monitoring of the harvested sites. Our results provide a quantitative basis for sustainable seed harvesting in wild populations. Yet, they are model results, and all models are simplifications of reality because it is impossible to capture the full complexity of the real world (Box, 1976). As a precaution, and to be able to adjust seed harvesting practice if necessary, it is therefore important to monitor the effects of the harvesting on wild populations. The safe seed fractions presented here cause only very slow population declines, maximum of 2% per year, and monitoring every few years should be sufficient to detect unexpected negative effects on population sizes before the population would be damaged irreversibly.

In summary, we found that seed harvesting in wild populations is possible and allows long‐term population persistence, but the harvesting strategy must be guided by the critical factors of the generation time of the species and the frequency of the harvesting. For longer‐lived species, harvesting large fractions of seeds is unlikely to harm wild populations, particularly if seeds are not harvested every year. For short‐lived species, though, more caution is necessary. A profitable harvesting of 30% of the seeds of annual species may be possible if the harvesting takes place only every 10 or more years. However, ultimately, even with improved guidelines, seed harvesting from wild populations is unlikely to cover the growing worldwide needs of ecological restoration (Pedrini et al., 2020). The ambitious targets of the UN Decade on Ecosystem Restoration https://www.decadeonrestoration.org/) may only be reached with professional, large‐scale seed production in seed orchards (Conrady et al., 2022, 2023; Espeland et al., 2017).

## Supporting information



Supporting Information

Supporting Information
