# Supplementary material for: Assessing limits of sustainable seed harvest in wild plant populations
Source: Conserv Biol. 2025 May 31;39(5):e70075. doi: 10.1111/cobi.70075 (PMC12451500; doi:10.1111/cobi.70075)
Supplement: Supplementary file 1 — Supporting Information [file COBI-39-e70075-s001.docx]

**Appendix S1**

**Assessing limits of sustainable seed harvest in wild plant populations**

Anna Bucharova, Oliver Bossdorf, J.F. Scheepens, Roberto Salguero-Gómez

Appendix S1. The final set of species used in this study and the original study that was the source of the MPMs.

| **Species** | **Publication** |
| --- | --- |
| *Abies concolor* | Van Mantgem, P. J., & Stephenson, N. L. (2005). The accuracy of matrix population model projections for coniferous trees in the Sierra Nevada, California. Journal of Ecology, 93(4), 737–747. Portico. https://doi.org/10.1111/j.1365-2745.2005.01007.x |
| *Abies magnifica* | Van Mantgem, P. J., & Stephenson, N. L. (2005). The accuracy of matrix population model projections for coniferous trees in the Sierra Nevada, California. Journal of Ecology, 93(4), 737–747. Portico. https://doi.org/10.1111/j.1365-2745.2005.01007.x |
| *Abies sachalinensis* | Hiura, T., & Fujiwara, K. (1999). Density‐dependence and co‐existence of conifer and broad‐leaved trees in a Japanese northern mixed forest. Journal of Vegetation Science, 10(6), 843–850. Portico. https://doi.org/10.2307/3237309 |
| *Acacia bilimekii* | Jiménez-Lobato, V., & Valverde, T. (2006). Population dynamics of the shrub *Acacia bilimekii* in a semi-desert region in central Mexico. Journal of Arid Environments, 65(1), 29–45. https://doi.org/10.1016/j.jaridenv.2005.07.002 |
| *Acacia suaveolens* | Warton, D. I., & Wardle, G. M. (2003). Site-to-site variation in the demography of a fire-affected perennial, *Acacia suaveolens*, at Ku-ring-gai Chase National Park, New South Wales, Australia. Austral Ecology, 28(1), 38–47. https://doi.org/10.1046/j.1442-9993.2003.01246.x |
| *Acer amoenum* | Tanaka, H., Shibata, M., Masaki, T., Iida, S., Niiyama, K., Abe, S., Kominami, Y., & Nakashizuka, T. (2008). Comparative demography of three coexisting *Acer* species in gaps and under closed canopy. Journal of Vegetation Science, 19(1), 127–138. Portico. https://doi.org/10.3170/2007-8-18342 |
| *Acer mono* | Tanaka, H., Shibata, M., Masaki, T., Iida, S., Niiyama, K., Abe, S., Kominami, Y., & Nakashizuka, T. (2008). Comparative demography of three coexisting *Acer* species in gaps and under closed canopy. Journal of Vegetation Science, 19(1), 127–138. Portico. https://doi.org/10.3170/2007-8-18342 |
| *Acer rufinerve* | Tanaka, H., Shibata, M., Masaki, T., Iida, S., Niiyama, K., Abe, S., Kominami, Y., & Nakashizuka, T. (2008). Comparative demography of three coexisting *Acer* species in gaps and under closed canopy. Journal of Vegetation Science, 19(1), 127–138. Portico. https://doi.org/10.3170/2007-8-18342 |
| *Acer saccharum* | Lin, Y., & Augspurger, C. K. (2008). Impact of spatial heterogeneity of neighborhoods on long-term population dynamics of sugar maple (*Acer saccharum*). Forest Ecology and Management, 255(10), 3589–3596. https://doi.org/10.1016/j.foreco.2008.02.040 |
| *Actaea spicata* | Fröborg, H., & Eriksson, O. (2003). Predispersal seed predation and population dynamics in the perennial understorey herb *Actaea spicata*. Canadian Journal of Botany, 81(11), 1058–1069. https://doi.org/10.1139/b03-099 |
| *Adesmia volckmannii* | Cipriotti, P. A., & Aguiar, M. R. (2011). Direct and indirect effects of grazing constrain shrub encroachment in semi-arid Patagonian steppes. Applied Vegetation Science, 15(1), 35–47. https://doi.org/10.1111/j.1654-109x.2011.01138.x |
| *Aeschynomene virginica* | Griffith, A. B., & Forseth, I. N. (2005). Population matrix models of *Aeschynomene virginica*, a rare annual plant: implications for conservation. Ecological Applications, 15(1), 222–233. https://doi.org/10.1890/02-5219 |
| *Aesculus turbinata* | Kaneko, Y., Takada, T., & Kawano, S. (1999). Population biology of *Aesculus turbinata* Blume: A demographic analysis using transition matrices on a natural population along a riparian environmental gradient. Plant Species Biology, 14(1), 47–68. https://doi.org/10.1046/j.1442-1984.1999.00007.x |
| *Agrimonia eupatoria* | Mondragón Chaparro, D., & Ticktin, T. (2011). Demographic effects of harvesting epiphytic bromeliads and an alternative approach to collection. Conservation Biology, 25(4), 797–807. https://doi.org/10.1111/j.1523-1739.2011.01691.x |
| *Ailanthus altissima* | Bullock, J. M., White, S. M., Prudhomme, C., Tansey, C., Perea, R., & Hooftman, D. A. P. (2011). Modelling spread of British wind-dispersed plants under future wind speeds in a changing climate. Journal of Ecology, 100(1), 104–115. https://doi.org/10.1111/j.1365-2745.2011.01910.x |
| *Alliaria petiolata* | Evans, J. A., Davis, A. S., Raghu, S., Ragavendran, A., Landis, D. A., & Schemske, D. W. (2012). The importance of space, time, and stochasticity to the demography and management of *Alliaria petiolata*. Ecological Applications, 22(5), 1497–1511. https://doi.org/10.1890/11-1291.1 |
| *Allium tricoccum* | Nault, A., & Gagnon, D. (1993). Ramet demography of *Allium tricoccum*, a spring ephemeral, perennial forest herb. The Journal of Ecology, 81(1), 101. https://doi.org/10.2307/2261228 |
| *Alyxia stellata* | Wong, T. M., & Ticktin, T. (2014). Using population dynamics modelling to evaluate potential success of restoration: a case study of a Hawaiian vine in a changing climate. Environmental Conservation, 42(1), 20–30. https://doi.org/10.1017/s0376892914000204 |
| *Andropogon brevifolius* | Canales, J., Trevisan, M.C., Silva, J.F. & Caswell,H. (1994): A demographic-study of an annual grass (*Andropogon brevifolius* Schwarz) in burnt and unburnt savanna. Acta Oecologica 15(3): 261-273 |
| *Androsace elongata* | Dostál, P. (2007). Population dynamics of annuals in perennial grassland controlled by ants and environmental stochasticity. Journal of Vegetation Science, 18(1), 91–102. Portico. https://doi.org/10.1111/j.1654-1103.2007.tb02519.x |
| *Anemone patens* | Williams, J. L., & Crone, E. E. (2006). The impact of invasive grasses on the population growth of *Anemone patens*, a long-lived native forb. Ecology, 87(12), 3200–3208. https://doi.org/10.1890/0012-9658(2006)87[3200:tioigo]2.0.co;2 |
| *Anthericum ramosum* | Černá, L., & Münzbergová, Z. (2013). Comparative population dynamics of two closely related species differing in ploidy level. PLoS ONE, 8(10), e75563. https://doi.org/10.1371/journal.pone.0075563 |
| *Anthyllis vulneraria* | Davison, R., Jacquemyn, H., Adriaens, D., Honnay, O., de Kroon, H., & Tuljapurkar, S. (2010). Demographic effects of extreme weather events on a short-lived calcareous grassland species: stochastic life table response experiments. Journal of Ecology, 98(2), 255–267. https://doi.org/10.1111/j.1365-2745.2009.01611.x |
| *Aquilaria crassna* | Zhang, L., Brockelman, W. Y., & Allen, M. A. (2008). Matrix analysis to evaluate sustainability: The tropical tree *Aquilaria crassna*, a heavily poached source of agarwood. Biological Conservation, 141(6), 1676–1686. https://doi.org/10.1016/j.biocon.2008.04.015 |
| *Aquilaria malaccensis* | Soehartono, T., & C. Newton, A. (2001). Conservation and sustainable use of tropical trees in the genus *Aquilaria* II. The impact of gaharu harvesting in Indonesia. Biological Conservation, 97(1), 29–41. https://doi.org/10.1016/s0006-3207(00)00089-6 |
| *Aquilaria microcarpa* | Soehartono, T., & C. Newton, A. (2001). Conservation and sustainable use of tropical trees in the genus *Aquilaria* II. The impact of gaharu harvesting in Indonesia. Biological Conservation, 97(1), 29–41. https://doi.org/10.1016/s0006-3207(00)00089-6 |
| *Aquilegia chrysantha* | Stubben, C.J. (2007). Projecting the response of yellow columbine populations to climate change. PhD thesis, New Mexico State University, Las Cruces, New Mexico. |
| *Aquilegia* sp*.* | Stubben, C. & Milligan, B. (2007): Estimating and analyzing demographic models using the popbio package in R. Journal of Statistics Software 22(11): 1-23 |
| *Araucaria araucana* | Bekessy, S., Newton, A., Fox, J., Lara, A., Premoli, A., Cortes, M., Gonzalez, M., & Burgman, M. (2004). Monkey puzzle tree (*Araucaria araucana*) in southern Chile: Effects of timber and seed harvest, volcanic activity, and fire. Species Conservation and Management: Case Studies (pp. 48-63). RMIT University. |
| *Araucaria cunninghamii* | Enright, N., & Ogden, J. (1979). Applications of transition matrix models in forest dynamics: *Araucaria* in Papua New Guinea and Nothofagus in New Zealand. Austral Ecology, 4(1), 3–23. https://doi.org/10.1111/j.1442-9993.1979.tb01195.x |
| *Araucaria hunsteinii* | Enright, N. J. (1982). Does *Araucaria hunsteinii* compete with its neighbours? Austral Ecology, 7(1), 97–99. https://doi.org/10.1111/j.1442-9993.1982.tb01304.x |
| *Araucaria muelleri* | Enright, N. J., Miller, B. P., Perry, G. L. W., Goldblum, D., & Jaffré, T. (2013). Stress-tolerator leaf traits determine population dynamics in the endangered New Caledonian conifer *Araucaria muelleri*. Austral Ecology, 39(1), 60–71. https://doi.org/10.1111/aec.12045 |
| *Arenaria bolosii* | Iriondo, J.M., Albert M. J., Gimenez-Benavides, L., Dominguez-Lozano, F. & Escudero, A. [Eds.] (2009): Poblaciones en peligro: viabilidad demográfica de la flora vascular amenazada de España. Dirección General de Medio Natural y Política Forestal (Ministerio de Medio Ambiente, y Medio Rural y Marino), Madrid, 242 pp. |
| *Arenaria serpyllifolia* | Dostál, P. (2007). Population dynamics of annuals in perennial grassland controlled by ants and environmental stochasticity. Journal of Vegetation Science, 18(1), 91–102. Portico. https://doi.org/10.1111/j.1654-1103.2007.tb02519.x |
| *Argyroxiphium sandwicense* subsp. *macrocephalum* | Forsyth, S. A. (2003). Density-dependent seed set in the *Haleakala* *silversword*: evidence for an Allee effect. Oecologia, 136(4), 551–557. https://doi.org/10.1007/s00442-003-1295-3 |
| *Armeria maritima* | Lefebvre, C., & Chandler-Mortimer, A. (1984). Demographic characteristics of the perennial herb *Armeria maritima* on zinc lead mine wastes. The Journal of Applied Ecology, 21(1), 255. https://doi.org/10.2307/2403051 |
| *Armeria merinoi* | Iriondo, J.M., Albert M. J., Gimenez-Benavides, L., Dominguez-Lozano, F. & Escudero, A. [Eds.] (2009): Poblaciones en peligro: viabilidad demográfica de la flora vascular amenazada de España. Dirección General de Medio Natural y Política Forestal (Ministerio de Medio Ambiente, y Medio Rural y Marino), Madrid, 242 pp. |
| *Artemisia genipi* | Marcante, S., Winkler, E., & Erschbamer, B. (2009). Population dynamics along a primary succession gradient: do alpine species fit into demographic succession theory? Annals of Botany, 103(7), 1129–1143. https://doi.org/10.1093/aob/mcp047 |
| *Asarum canadense* | Damman, H., & Cain, M. L. (1998). Population growth and viability analyses of the clonal woodland herb, *Asarum canadense*. Journal of Ecology, 86(1), 13–26. https://doi.org/10.1046/j.1365-2745.1998.00242.x |
| *Astragalus alopecurus* | Nicole F. (2005): Biologie de la conservation appliquée aux plantes menacées des Alpes. PhD thesis, University of Grenoble, Grenoble, France. |
| *Astragalus australis* var. *olympicus* | Kaye, T. (1990). Autecology, reproductive ecology, and demography of *Astragalus australis* var. *olympicus* (Fabaceae). MSc. Thesis, Oregon State University. |
| *Astragalus peckii* | Martin, E. F., & Meinke, R. J. (2012). Variation in the demographics of a rare central Oregon endemic, *Astragalus peckii* Piper (Fabaceae), with fluctuating levels of herbivory. Population Ecology, 54(3), 381–390. Portico. https://doi.org/10.1007/s10144-012-0318-5 |
| *Astragalus scaphoides* | Lesica, P (1995): Demography of *Astragalus scaphoides* and effects of herbivory on population-growth. Great Basin Naturalis 55(2): 142-150 |
| *Astragalus tyghensis* | Kaye, T. N., & Pyke, D. A. (2003). The effect of stochastic technique on estimates of population viability from transition matrix models. Ecology, 84(6), 1464–1476. https://doi.org/10.1890/0012-9658(2003)084[1464:teosto]2.0.co;2 |
| *Astrophytum asterias* | Martínez-Ávalos J.G. (2007): Estudio demográfico del “star cactus” *Astrophytum asterias* (Lem.) Zucc. (Cactaceae) una especie en riesgo de extinción. PhD Thesis, Universidad Autónoma de Nuevo León, México. |
| *Astrophytum capricorne* | Mandujano, M. C., Bravo, Y., Verhulst, J., Carrillo-Angeles, I., & Golubov, J. (2015). The population dynamics of an endemic collectible cactus. Acta Oecologica, 63, 1–7. https://doi.org/10.1016/j.actao.2014.12.004 |
| *Astrophytum ornatum* | Zepeda-Martínez, V., Mandujano, M. C., Mandujano, F. J., & Golubov, J. K. (2013). What can the demography of *Astrophytum ornatum* tell us of its endangered status? Journal of Arid Environments, 88, 244–249. https://doi.org/10.1016/j.jaridenv.2012.08.006 |
| *Avicennia germinans* | López-Hoffman, L., Ackerly, D. D., Anten, N. P. R., Denoyer, J. L., & Martinez-Ramos, M. (2007). Gap-dependence in mangrove life-history strategies: a consideration of the entire life cycle and patch dynamics. Journal of Ecology, 95(6), 1222–1233. https://doi.org/10.1111/j.1365-2745.2007.01298.x |
| *Banksia ericifolia* | Bradstock, R. A., & O’Connell, M. A. (1988). Demography of woody plants in relation to fire: *Banksia ericifolia* L.f. and *Petrophile pulchella* (Schrad) R.Br. Austral Ecology, 13(4), 505–518. https://doi.org/10.1111/j.1442-9993.1988.tb00999.x |
| *Borassus aethiopum* | Barot, S., Gignoux, J., Vuattoux, R., & Legendre, S. (2000). Demography of a savanna palm tree in Ivory Coast (Lamto): population persistence and life-history. Journal of Tropical Ecology, 16(5), 637–655. https://doi.org/10.1017/s0266467400001620 |
| *Borderea chouardii* | Garcı́a, M. B., Guzmán, D., & Goñi, D. (2002). An evaluation of the status of five threatened plant species in the Pyrenees. Biological Conservation, 103(2), 151–161. https://doi.org/10.1016/s0006-3207(01)00113-6 |
| *Boswellia papyrifera* | Groenendijk, P., Eshete, A., Sterck, F. J., Zuidema, P. A., & Bongers, F. (2011). Limitations to sustainable frankincense production: blocked regeneration, high adult mortality and declining populations. Journal of Applied Ecology, 49(1), 164–173. Portico. https://doi.org/10.1111/j.1365-2664.2011.02078.x |
| *Brassica insularis* | Noël, F., Maurice, S., Mignot, A., Glémin, S., Carbonell, D., Justy, F., Guyot, I., Olivieri, I., & Petit, C. (2010). Interaction of climate, demography and genetics: a ten-year study of *Brassica insularis*, a narrow endemic Mediterranean species. Conservation Genetics, 11(2), 509–526. https://doi.org/10.1007/s10592-010-0056-1 |
| *Brassica napus* | Garnier, A., & Lecomte, J. (2006). Using a spatial and stage-structured invasion model to assess the spread of feral populations of transgenic oilseed rape. Ecological Modelling, 194(1–3), 141–149. https://doi.org/10.1016/j.ecolmodel.2005.10.009 |
| *Braya fernaldii* | Squires, S. (2010): Insect pests and pathogens compromise the persistence of two endemic and rare *Braya* (Brassicaceae). PhD Dissertation, memorial University of Newfoundland, Newfoundland and labrador, 184 pp. |
| *Braya longii* | Squires, S. (2010): Insect pests and pathogens compromise the persistence of two endemic and rare *Braya* (Brassicaceae). PhD Dissertation, memorial University of Newfoundland, Newfoundland and Labrador, 184 pp. |
| *Bursera glabrifolia* | Hernández-Apolinar, M., Valverde, T., & Purata, S. (2006). Demography of *Bursera glabrifolia*, a tropical tree used for folk woodcrafting in Southern Mexico: An evaluation of its management plan. Forest Ecology and Management, 223(1–3), 139–151. https://doi.org/10.1016/j.foreco.2005.10.072 |
| *Calathea ovandensis* | Horvitz, C. C., & Schemske, D. W. (1995). Spatiotemporal variation in demographic transitions of a tropical understory herb: projection matrix analysis. Ecological Monographs, 65(2), 155–192. Portico. https://doi.org/10.2307/2937136 |
| *Callitris intratropica* | Price, O., & Bowman, D. M. J. S. (1994). Fire-stick forestry: a matrix model in support of skillful fire management of *Callitris intratropica* R. T. Baker by north Australian Aborigenes. Journal of Biogeography, 21(6), 573. https://doi.org/10.2307/2846032 |
| *Calluna vulgaris* | Scandrett, E., & Gimingham, C. H. (1989). A model of *Calluna* population dynamics; the effects of varying seed and vegetative regeneration. Vegetatio, 84(2), 143–152. https://doi.org/10.1007/bf00036515 |
| *Calocedrus decurrens* | Van Mantgem, P. J., & Stephenson, N. L. (2005). The accuracy of matrix population model projections for coniferous trees in the Sierra Nevada, California. Journal of Ecology, 93(4), 737–747. Portico. https://doi.org/10.1111/j.1365-2745.2005.01007.x |
| *Calochortus albus* | Fiedler, P. L. (1987). Life history and population dynamics of rare and common mariposa lilies (*Calochortus pursh*: Liliaceae). The Journal of Ecology, 75(4), 977. https://doi.org/10.2307/2260308 |
| *Calochortus obispoensis* | Fiedler, P. L. (1987). Life history and population dynamics of rare and common mariposa lilies (*Calochortus pursh*: Liliaceae). The Journal of Ecology, 75(4), 977. https://doi.org/10.2307/2260308 |
| *Calochortus pulchellus* | Fiedler, P. L. (1987). Life history and population dynamics of rare and common mariposa lilies (*Calochortus pursh*: Liliaceae). The Journal of Ecology, 75(4), 977. https://doi.org/10.2307/2260308 |
| *Calochortus tiburonensis* | Fiedler, P. L. (1987). Life history and population dynamics of rare and common mariposa lilies (*Calochortus pursh*: Liliaceae). The Journal of Ecology, 75(4), 977. https://doi.org/10.2307/2260308 |
| *Carduus nutans* | Shea, K., Kelly, D., Sheppard, A. W., & Woodburn, T. L. (2005). Context-dependent biological control of an invasive thistle. Ecology, 86(12), 3174–3181. https://doi.org/10.1890/05-0195 |
| *Carlina vulgaris* | Jongejans, E., Jorritsma-Wienk, L. D., Becker, U., Dostál, P., Mildén, M., & de Kroon, H. (2010). Region versus site variation in the population dynamics of three short-lived perennials. Journal of Ecology, 98(2), 279–289. https://doi.org/10.1111/j.1365-2745.2009.01612.x |
| *Carnegiea gigantea* | Steenbergh, W. F., & Lowe, C. H. (1969). Critical factors during the first years of life of the saguaro (*Cereus giganteus*) at Saguaro National Monument, Arizona. Ecology, 50(5), 825–834. Portico. https://doi.org/10.2307/1933696 |
| *Cassia nemophila* | Silander, J. A. (1983). Demographic variation in the Australian desert cassia under grazing pressure. Oecologia, 60(2), 227–233. https://doi.org/10.1007/bf00379524 |
| *Castanea dentata* | Davelos, A. L., & Jarosz, A. M. (2004). Demography of American chestnut populations: effects of a pathogen and a hyperparasite. Journal of Ecology, 92(4), 675–685. Portico. https://doi.org/10.1111/j.0022-0477.2004.00907.x |
| *Cecropia obtusifolia* | Alvarez-Buylla, E. R. (1994). Density dependence and patch dynamics in tropical rain forests: matrix models and applications to a tree species. The American Naturalist, 143(1), 155–191. https://doi.org/10.1086/285599 |
| *Centaurea maculosa* | Emery, S. M., & Gross, K. L. (2005). Effects of timing of prescribed fire on the demography of an invasive plant, spotted knapweed *Centaurea maculosa*. Journal of Applied Ecology, 42(1), 60–69. https://doi.org/10.1111/j.1365-2664.2004.00990.x |
| *Cephalanthera longifolia* | Shefferson, R. P., Kull, T., Tali, K., & Kellett, K. M. (2012). Linking vegetative dormancy to fitness in two long-lived herbaceous perennials. Ecosphere, 3(2), art13. https://doi.org/10.1890/es11-00328.1 |
| *Chaerophyllum aureum* | Magda, D., Duru, M., & Theau, J.-P. (2004). Defining management rules for grasslands using weed demographic characteristics. Weed Science, 52(3), 339–345. https://doi.org/10.1614/p2202-067 |
| *Chamaecrista keyensis* | Liu, H., Menges, E. S., & Quintana-Ascencio, P. F. (2005). Population viability analyses of *Chamaecrista keyensis*: Effects of fire season and frequency. Ecological Applications, 15(1), 210–221. https://doi.org/10.1890/03-5382 |
| *Chamaedorea elegans* | Valverde, T., Hernandez-Apolinar, M., & Mendoza-Amarom, S. (2006). Effect of leaf harvesting on the demography of the tropical palm *Chamaedorea elegans* in South-Eastern Mexico. Journal of Sustainable Forestry, 23(1), 85–105. https://doi.org/10.1300/j091v23n01_05 |
| *Chamaedorea radicalis* | Endress, B. A., Gorchov, D. L., & Noble, R. B. (2004).Non-timber forest product extraction: effects of harvest and browsing on an understory palm. Ecological Applications, 14(4), 1139–1153. https://doi.org/10.1890/02-5365 |
| *Choerospondias axillaris* | Brodie, J. F., Helmy, O. E., Brockelman, W. Y., & Maron, J. L. (2009). Functional differences within a guild of tropical mammalian frugivores. Ecology, 90(3), 688–698. Portico. https://doi.org/10.1890/08-0111.1 |
| *Cimicifuga elata* | Kaye, T. N., & Pyke, D. A. (2003). The effect of stochastic technique on estimates of population viability from transition matrix models. Ecology, 84(6), 1464–1476. https://doi.org/10.1890/0012-9658(2003)084[1464:teosto]2.0.co;2 |
| *Cirsium acaule* | Bullock, J. M., White, S. M., Prudhomme, C., Tansey, C., Perea, R., & Hooftman, D. A. P. (2011). Modelling spread of British wind-dispersed plants under future wind speeds in a changing climate. Journal of Ecology, 100(1), 104–115. https://doi.org/10.1111/j.1365-2745.2011.01910.x |
| *Cirsium dissectum* | Jongejans, E., de Vere, N., & de Kroon, H. (2008). Demographic vulnerability of the clonal and endangered meadow thistle. Plant Ecology, 198(2), 225–240. https://doi.org/10.1007/s11258-008-9397-y |
| *Cirsium palustre* | Ramula, S. (2008). Population dynamics of a monocarpic thistle: simulated effects of reproductive timing and grazing of flowering plants. Acta Oecologica, 33(2), 231–239. https://doi.org/10.1016/j.actao.2007.11.005 |
| *Cirsium perplexans* | Dodge, G.J. 2005. Ecological effects of the biocontrol insects, *Larinus planus* and *Rhinocyllus conicus*, on native thistles. PhD Thesis, University of Maryland, Maryland |
| *Cirsium scariosum* | Dodge, G.J. 2005. Ecological effects of the biocontrol insects, *Larinus planus* and *Rhinocyllus conicus*, on native thistles. PhD Thesis, University of Maryland, Maryland |
| *Cirsium undulatum* var. *tracyi* | Dodge, G.J. 2005. Ecological effects of the biocontrol insects, *Larinus planus* and *Rhinocyllus conicus*, on native thistles. PhD Thesis, University of Maryland, Maryland |
| *Cirsium vulgare* | Bullock, J. M., Hill, B. C., & Silvertown, J. (1994). Demography of *Cirsium vulgare* in a grazing experiment. The Journal of Ecology, 82(1), 101. https://doi.org/10.2307/2261390 |
| *Cochlearia bavarica* | Abs, C. (1999). Differences in the life histories of two *Cochlearia species*. Folia Geobotanica, 34(1), 33–45. https://doi.org/10.1007/bf02803075 |
| *Cochlearia pyrenaica* | Abs, C. (1999). Differences in the life histories of two *Cochlearia species*. Folia Geobotanica, 34(1), 33–45. https://doi.org/10.1007/bf02803075 |
| *Colchicum autumnale* | Winter, S., Jung, L. S., Eckstein, R. L., Otte, A., Donath, T. W., & Kriechbaum, M. (2014). Control of the toxic plant *Colchicum autumnale* in semi-natural grasslands: effects of cutting treatments on demography and diversity. Journal of Applied Ecology, 51(2), 524–533. https://doi.org/10.1111/1365-2664.12217 |
| *Collinsia verna* | Kalisz, S., & McPeek, M. A. (1992). Demography of an age-structured annual: resampled projection matrices, elasticity analyses, and seed bank effects. Ecology, 73(3), 1082–1093. Portico. https://doi.org/10.2307/1940182 |
| *Conyza canadensis* | Bullock, J. M., White, S. M., Prudhomme, C., Tansey, C., Perea, R., & Hooftman, D. A. P. (2011). Modelling spread of British wind-dispersed plants under future wind speeds in a changing climate. Journal of Ecology, 100(1), 104–115. https://doi.org/10.1111/j.1365-2745.2011.01910.x |
| *Coryphantha robbinsorum* | Schmalzel, R.J., Reichenbacher F.W. & Rutman S. (1995). Demographic study of the rare *Coryphantha robbinsorum* (Cactaceae) in southeastern Arizona. Madroño 42: 332-348 |
| *Cynoglossum officinale* | Boorman, L. A., & Fuller, R. M. (1984). The comparative ecology of two sand dune biennials: *Lactuca virosa* L. and *Cynoglossum officinale* L. New Phytologist, 96(4), 609–629. https://doi.org/10.1111/j.1469-8137.1984.tb03596.x |
| *Cypripedium calceolus* | Nicolè, F., Brzosko, E., & Till-Bottraud, I. (2005). Population viability analysis of *Cypripedium calceolus* in a protected area: longevity, stability and persistence. Journal of Ecology, 93(4), 716–726. Portico. https://doi.org/10.1111/j.1365-2745.2005.01010.x |
| *Cypripedium parviflorum* var. *parviflorum* | Shefferson, R. P., Warren, R. J., & Pulliam, H. R. (2014). Life-history costs make perfect sprouting maladaptive in two herbaceous perennials. Journal of Ecology, 102(5), 1318–1328. https://doi.org/10.1111/1365-2745.12281 |
| *Cytisus scoparius* | da Silveira Pontes, L., Magda, D., Jarry, M., Gleizes, B., & Agreil, C. (2012). Shrub encroachment control by browsing: Targeting the right demographic process. Acta Oecologica, 45, 25–30. https://doi.org/10.1016/j.actao.2012.08.006 |
| *Dactylorhiza lapponica* | Sletvold, N., Øien, D.-I., & Moen, A. (2010). Long-term influence of mowing on population dynamics in the rare orchid *Dactylorhiza lapponica*: The importance of recruitment and seed production. Biological Conservation, 143(3), 747–755. https://doi.org/10.1016/j.biocon.2009.12.017 |
| *Daphne rodriguezii* | Rodríguez-Ortega C. (2008). Consecuencias demográficas y evolutivas del secuestro de semillas en tres especies del género *Mammillaria* (Cactaceae). PhD Dissertation, Universidad Autónoma Metropolitana, Mexico. |
| *Daucus carota* | Verkaar, H. J., & Schenkeveld, A. J. (1984). On the ecology of short-lived forbs in chalk grasslands: life-history characteristics. New Phytologist, 98(4), 659–672. https://doi.org/10.1111/j.1469-8137.1984.tb04155.x |
| *Dicerandra frutescens* | Menges, E. S., Quintana Ascencio, P. F., Weekley, C. W., & Gaoue, O. G. (2006). Population viability analysis and fire return intervals for an endemic Florida scrub mint. Biological Conservation, 127(1), 115–127. https://doi.org/10.1016/j.biocon.2005.08.002 |
| *Digitalis purpurea* | van Baalen, J., & Prins, E. G. M. (1983). Growth and reproduction of *Digitalis purpurea* in different stages of succession. Oecologia, 58(1), 84–91. https://doi.org/10.1007/bf00384546 |
| *Dioon merolae* | Lázaro-Zermeño, J. M., González-Espinosa, M., Mendoza, A., Martínez-Ramos, M., & Quintana-Ascencio, P. F. (2011). Individual growth, reproduction and population dynamics of *Dioon merolae* (Zamiaceae) under different leaf harvest histories in Central Chiapas, Mexico. Forest Ecology and Management, 261(3), 427–439. https://doi.org/10.1016/j.foreco.2010.10.028 |
| *Dipsacus sylvestris* | Werner, P. A., & Caswell, H. (1977). Population rowth rates and age versus stage-distribution models for teasel (*Dipsacus sylvestris* Huds.). Ecology, 58(5), 1103–1111. Portico. https://doi.org/10.2307/1936930 |
| *Draba asterophora* | Putnam, E. R. S. (2013): Ecology, phylogenetics, and conservation of *Draba asterophora* complex: a rare, alpine, endemic from Lake Tahoe, USA. PhD Thesis, Birgham Young University, Utah. |
| *Dracocephalum austriacum* | Dostálek, T., & Münzbergová, Z. (2012).Comparative population biology of critically endangered *Dracocephalum austriacum* (Lamiaceae) in two distant regions. Folia Geobotanica, 48(1), 75–93. https://doi.org/10.1007/s12224-012-9132-2 |
| *Echeveria longissima* | Martorell, C., Garcillán, P. P., & Casillas, F. (2012). Ruderality in extreme‐desert cacti? Population effects of chronic anthropogenic disturbance on *Echinocereus lindsayi*. Population Ecology, 54(2), 335–346. Portico. https://doi.org/10.1007/s10144-012-0307-8 |
| *Echinocactus platyacanthus* | Jiménez-Sierra, C., Mandujano, M. C., & Eguiarte, L. E. (2007). Are populations of the candy barrel cactus (*Echinocactus platyacanthus*) in the desert of Tehuacán, Mexico at risk? Population projection matrix and life table response analysis. Biological Conservation, 135(2), 278–292. https://doi.org/10.1016/j.biocon.2006.10.038 |
| *Echinospartum algibicum* | Iriondo, J.M., Albert M. J., Gimenez-Benavides, L., Dominguez-Lozano, F. & Escudero, A. [Eds.] (2009): Poblaciones en peligro: viabilidad demográfica de la flora vascular amenazada de España. Dirección General de Medio Natural y Política Forestal (Ministerio de Medio Ambiente, y Medio Rural y Marino), Madrid, 242 pp. |
| *Echium vulgare* | Klemow, K. M., & Raynal, D. J. (1985). Demography of two facultative biennial plant species in an unproductive habitat. The Journal of Ecology, 73(1), 147. https://doi.org/10.2307/2259775 |
| *Encephalartos cycadifolius* | Raimondo, D. C., & Donaldson, J. S. (2003). Responses of cycads with different life histories to the impact of plant collecting: simulation models to determine important life history stages and population recovery times. Biological Conservation, 111(3), 345–358. https://doi.org/10.1016/s0006-3207(02)00303-8 |
| *Epipactis atrorubens* | Hens, H., Pakanen, V.-M., Jäkäläniemi, A., Tuomi, J., & Kvist, L. (2017). Low population viability in small endangered orchid populations: Genetic variation, seedling recruitment and stochasticity. Biological Conservation, 210, 174–183. https://doi.org/10.1016/j.biocon.2017.04.019 |
| *Eriogonum longifolium* var. *gnaphalifolium* | Satterthwaite, W. H., Menges, E. S., & Quintana-Ascencio, P. F. (2002). Assessing scrub buckwheat population viability in relation to fire using multiple modeling techniques. Ecological Applications, 12(6), 1672–1687. https://doi.org/10.1890/1051-0761(2002)012[1672:asbpvi]2.0.co;2 |
| *Eryngium alpinum* | Andrello, M., Bizoux, J.-P., Barbet-Massin, M., Gaudeul, M., Nicolè, F., & Till-Bottraud, I. (2012). Effects of management regimes and extreme climatic events on plant population viability in *Eryngium alpinum*. Biological Conservation, 147(1), 99–106. https://doi.org/10.1016/j.biocon.2011.12.012 |
| *Eryngium cuneifolium* | Menges, E. S., & Quintana-Ascencio, P. F. (2004). Population viability with fire in *Eryngium cuneifolium*: Deciphering a decade of demographic data. Ecological Monographs, 74(1), 79–99. https://doi.org/10.1890/03-4029 |
| *Erythronium japonicum* | Kawano S., Takada T., Nakayama S., Hiratsuka A. (1987) Demographic differentiation and life-history evolution in temperate woodland plants. In: Urbanska K. M. (ed.). Differentiation Patterns in Higher Plants. Academic Press, Harcourt Brace Jovanovich Publishers, London, pp. 153– 181. |
| *Escontria chiotilla* | Ortega, B. (2001). Demografía de la cactácea columnar *Escontria chiotilla*. MSc. Thesis, Universidad Autónoma Metropolitana, Mexico. |
| *Espeletia spicata* | Silva, J. F., Trevisan, M. C., Estrada, C. A., & Monasterio, M. (2000). Comparative demography of two giant caulescent rosettes (*Espeletia timotensis* and *E. spicata*) from the high tropical Andes. Global Ecology and Biogeography, 9(5), 403–413. Portico. https://doi.org/10.1046/j.1365-2699.2000.00187.x |
| *Espeletia timotensis* | Silva, J. F., Trevisan, M. C., Estrada, C. A., & Monasterio, M. (2000). Comparative demography of two giant caulescent rosettes (*Espeletia timotensis* and *E. spicata*) from the high tropical Andes. Global Ecology and Biogeography, 9(5), 403–413. Portico. https://doi.org/10.1046/j.1365-2699.2000.00187.x |
| *Eupatorium perfoliatum* | Byers, D. L., & Meagher, T. R. (1997).A comparison of demographic characteristics in a rare and a common species of Eupatorium. Ecological Applications, 7(2), 519–530. https://doi.org/10.1890/1051-0761(1997)007[0519:acodci]2.0.co;2 |
| *Eupatorium resinosum* | Byers, D. L., & Meagher, T. R. (1997).A comparison of demographic characteristics in a rare and a common species of *Eupatorium*. Ecological Applications, 7(2), 519–530. https://doi.org/10.1890/1051-0761(1997)007[0519:acodci]2.0.co;2 |
| *Fagus grandifolia* | Batista, W. B., Platt, W. J., & Macchiavelli, R. E. (1998). Demography of a shade-tolerant tree (*Fagus grandifolia*) in a hurricane-disturbed forest. Ecology, 79(1), 38. https://doi.org/10.2307/176863 |
| *Fritillaria meleagris* | Zhang, L., & Hytteborn, H. (1985). Effect of ground water regime on development and distribution of *Fritillaria meleagri*s. Ecography, 8(4), 237–244. https://doi.org/10.1111/j.1600-0587.1985.tb01174.x |
| *Gardenia actinocarpa* | Osunkoya, O. O. (2003). Two-sex population projection of the endemic and dioecious rainforest shrub, *Gardenia actinocarpa* (Rubiaceae). Biological Conservation, 114(1), 39–51. https://doi.org/10.1016/s0006-3207(02)00417-2 |
| *Gentianella campestris* | Lennartsson, T., & Oostermeijer, J. G. B. (2001). Demographic variation and population viability in *Gentianella campestris*: effects of grassland management and environmental stochasticity. Journal of Ecology, 89(3), 451–463. Portico. https://doi.org/10.1046/j.1365-2745.2001.00566.x |
| *Geonoma brevispatha* | Souza, A. F., & Martins, F. R. (2006). Demography of the clonal palm *Geonoma brevispatha* in a Neotropical swamp forest. Austral Ecology, 31(7), 869–881. https://doi.org/10.1111/j.1442-9993.2006.01650.x |
| *Geonoma orbignyana* | Rodríguez-Buriticá, S., Orjuela, M. A., & Galeano, G. (2005). Demography and life history of *Geonoma orbignyana*: An understory palm used as foliage in Colombia. Forest Ecology and Management, 211(3), 329–340. https://doi.org/10.1016/j.foreco.2005.02.052 |
| *Geonoma schottiana* | Sampaio, M. B., & Scariot, A. (2010). Effects of stochastic herbivory events on population maintenance of an understorey palm species (*Geonoma schottiana*) in riparian tropical forest. Journal of Tropical Ecology, 26(2), 151–161. https://doi.org/10.1017/s0266467409990599 |
| *Geranium sylvaticum* | Ramula, S., Toivonen, E., & Mutikainen, P. (2007). Demographic consequences of pollen limitation and inbreeding depression in a gynodioecious herb. International Journal of Plant Sciences, 168(4), 443–453. https://doi.org/10.1086/512040 |
| *Geum rivale* | Kiviniemi, K. (2002). Population dynamics of *Agrimonia eupatoria* and *Geum rivale*, two perennial grassland species. Plant Ecology, 159(2), 153–169. https://doi.org/10.1023/a:1015506019670 |
| *Gilia tenuiflora subsp. hoffmannii* | Levine, J. M., McEachern, A. K., & Cowan, C. (2008). Rainfall effects on rare annual plants. Journal of Ecology, 96(4), 795–806. https://doi.org/10.1111/j.1365-2745.2008.01375.x |
| *Grias peruviana* | Peters, C.M. 1990b. Population ecology and management of forest fruit trees in Peruvian Amazonian. In A.B. Anderson (ed.), Alternatives to Deforestation: Steps Toward Sustainable Use of the Amazon Rain Forest, pp. 86-98. Columbia University Press, New York |
| *Guaiacum sanctum* | Lopez-Toledo, L., Burslem, D. F. R. P., Martinez-Ramos, M. & Garcia-Naranno, A. (2008): Non-detriment findings report on *Guaiacum sanctum* in Mexico. NDF Workshop Case Studies, WG1 - Trees, Case Study 7. CITES Plant Commitee, Mexico |
| *Haplopappus radiatus* | Kaye, T. N., & Pyke, D. A. (2003). The effect of stochastic technique on estimates of population viability from transition matrix models. Ecology, 84(6), 1464–1476. https://doi.org/10.1890/0012-9658(2003)084[1464:teosto]2.0.co;2 |
| *Helenium virginicum* | Adams, V. M., Marsh, D. M., & Knox, J. S. (2005). Importance of the seed bank for population viability and population monitoring in a threatened wetland herb. Biological Conservation, 124(3), 425–436. https://doi.org/10.1016/j.biocon.2005.02.001 |
| *Helianthemum juliae* | Marrero-Gómez, M. V., Oostermeijer, J. G. B., Carqué-Álamo, E., & Bañares-Baudet, Á. (2007). Population viability of the narrow endemic *Helianthemum juliae* (Cistaceae) in relation to climate variability. Biological Conservation, 136(4), 552–562. https://doi.org/10.1016/j.biocon.2007.01.010 |
| *Helianthemum polygonoides* | Iriondo, J.M., Albert M. J., Gimenez-Benavides, L., Dominguez-Lozano, F. & Escudero, A. [Eds.] (2009): Poblaciones en peligro: viabilidad demográfica de la flora vascular amenazada de España. Dirección General de Medio Natural y Política Forestal (Ministerio de Medio Ambiente, y Medio Rural y Marino), Madrid, 242 pp. |
| *Heliconia acuminata* | Bruna, E. M. (2003). Are plant populations in fragmented habitats recruitment limited? Tests with an Amazonian herb. Ecology, 84(4), 932–947. https://doi.org/10.1890/0012-9658(2003)084[0932:appifh]2.0.co;2 |
| *Heracleum mantegazzianum* | Nehrbass, N., Winkler, E., Pergl, J., Perglova, I., & Pysek, P. (2006). Empirical and virtual investigation of the population dynamics of an alien plant under the constraints of local carrying capacity: *Heracleum mantegazzianum* in the Czech Republic. Perspectives in Plant Ecology, Evolution and Systematics, 7(4), 253–262. https://doi.org/10.1016/j.ppees.2005.11.001 |
| *Hieracium floribundum* | Thomas, A. G., & Dale, H. M. (1975). The role of seed reproduction in the dynamics of established populations of *Hieracium floribundum* and a comparison with that of vegetative reproduction. Canadian Journal of Botany, 53(24), 3022–3031. https://doi.org/10.1139/b75-331 |
| *Himantoglossum hircinum* | Pfeifer, M., Wiegand, K., Heinrich, W., & Jetschke, G. (2006). Long-term demographic fluctuations in an orchid species driven by weather: implications for conservation planning. Journal of Applied Ecology, 43(2), 313–324. https://doi.org/10.1111/j.1365-2664.2006.01148.x |
| *Hymenoxys herbacea* | Campbell, L. G., & Husband, B. C. (2005). Impact of clonal growth on effective population size in *Hymenoxys herbacea* (Asteraceae). Heredity, 94(5), 526–532. https://doi.org/10.1038/sj.hdy.6800653 |
| *Hypericum cumulicola* | Quintana-Ascencio, P. F., Menges, E. S., & Weekley, C. W. (2003). A fire-explicit population viability analysis of *Hypericum cumulicola* in Florida rosemary scrub. Conservation Biology, 17(2), 433–449. https://doi.org/10.1046/j.1523-1739.2003.01431.x |
| *Ipomopsis tenuituba* | Campbell, D. R., & Waser, N. M. (2007). Evolutionary dynamics of an *Ipomopsis* hybrid zone: Confronting models with lifetime fitness data. The American Naturalist, 169(3), 298–310. https://doi.org/10.1086/510758 |
| *Iris germanica* | Burns, J. H., Pardini, E. A., Schutzenhofer, M. R., Chung, Y. A., Seidler, K. J., & Knight, T. M. (2013). Greater sexual reproduction contributes to differences in demography of invasive plants and their noninvasive relatives. Ecology, 94(5), 995–1004. https://doi.org/10.1890/12-1310.1 |
| *Jurinea fontqueri* | Iriondo, J.M., Albert M. J., Gimenez-Benavides, L., Dominguez-Lozano, F. & Escudero, A. [Eds.] (2009): Poblaciones en peligro: viabilidad demográfica de la flora vascular amenazada de España. Dirección General de Medio Natural y Política Forestal (Ministerio de Medio Ambiente, y Medio Rural y Marino), Madrid, 242 pp. |
| *Khaya senegalensis* | Gaoue, O. G., & Ticktin, T. (2010). Effects of harvest of nontimber forest products and ecological differences between sites on the demography of african mahogany. Conservation Biology, 24(2), 605–614. https://doi.org/10.1111/j.1523-1739.2009.01345.x |
| *Knautia arvensis* | Johansen, L., Wehn, S., & Hovstad, K. A. (2016). Clonal growth buffers the effect of grazing management on the population growth rate of a perennial grassland herb. Flora, 223, 11–18. https://doi.org/10.1016/j.flora.2016.04.007 |
| *Kosteletzkya pentacarpos* | Pino, J., Picó, F. X., & De Roa, E.(2007). Population dynamics of the rare plant *Kosteletzkya pentacarpos* (Malvaceae): a nine‐year study. Botanical Journal of the Linnean Society, 153(4), 455–462. https://doi.org/10.1111/j.1095-8339.2007.00628.x |
| *Kummerowia striata* | Levin, S. C., Crandall, R. M., & Knight, T. M. (2019). Population projection models for 14 alien plant species in the presence and absence of aboveground competition. Ecology, e02681. Portico. https://doi.org/10.1002/ecy.2681 |
| *Lactuca serriola* | Bullock, J. M., White, S. M., Prudhomme, C., Tansey, C., Perea, R., & Hooftman, D. A. P. (2011). Modelling spread of British wind-dispersed plants under future wind speeds in a changing climate. Journal of Ecology, 100(1), 104–115. https://doi.org/10.1111/j.1365-2745.2011.01910.x |
| *Lantana camara* | Raghu, S., Osunkoya, O. O., Perrett, C., & Pichancourt, J.-B. (2014). Historical demography of *Lantana camara* L. reveals clues about the influence of land use and weather in the management of this widespread invasive species. Basic and Applied Ecology, 15(7), 565–572. https://doi.org/10.1016/j.baae.2014.08.006 |
| *Lathyrus vernus* | de Vries, C., & Caswell, H. (2017). Demography when history matters: construction and analysis of second-order matrix population models. Theoretical Ecology, 11(2), 129–140. https://doi.org/10.1007/s12080-017-0353-0 |
| *Lathyrus vernus* | Ehrlen, J. (1995). Demography of the perennial herb *Lathyrus vernus*. II. Herbivory and population dynamics. The Journal of Ecology, 83(2), 297. https://doi.org/10.2307/2261568 |
| *Lechea cernua* | Maliakal Witt, S. (2004): Microhabitat distribution and demography of two Florida scrub endemic plants with comparisons to their habitat-generalist congeners. PhD Thesis, Louisiana State Eniversity, Louisiana. |
| *Lechea deckertii* | Maliakal Witt, S. (2004): Microhabitat distribution and demography of two Florida scrub endemic plants with comparisons to their habitat-generalist congeners. PhD Thesis, Louisiana State Eniversity, Louisiana. |
| *Leucopogon setiger* | Swab R. M. (2014): Increasing understanding of species responses to global changes through modeling plant metapopulation dynamics. PhD Thesis, University of California, Riverside. |
| *Limonium carolinianum* | Baltzer, J. L., Reekie, E. G., Hewlin, H. L., Taylor, P. D., & Boates, J. S. (2002). Impact of flower harvesting on the salt marsh plant *Limonium carolinianum*. Canadian Journal of Botany, 80(8), 841–851. https://doi.org/10.1139/b02-070 |
| *Limonium delicatulum* | Hegazy, A. K. (1992). Age-specific survival, mortality and reproduction, and prospects for conservation of *Limonium delicatulum*. The Journal of Applied Ecology, 29(3), 549. https://doi.org/10.2307/2404462 |
| *Limonium erectum* | Iriondo, J.M., Albert M. J., Gimenez-Benavides, L., Dominguez-Lozano, F. & Escudero, A. [Eds.] (2009): Poblaciones en peligro: viabilidad demográfica de la flora vascular amenazada de España. Dirección General de Medio Natural y Política Forestal (Ministerio de Medio Ambiente, y Medio Rural y Marino), Madrid, 242 pp. |
| *Limonium geronense* | Iriondo, J.M., Albert M. J., Gimenez-Benavides, L., Dominguez-Lozano, F. & Escudero, A. [Eds.] (2009): Poblaciones en peligro: viabilidad demográfica de la flora vascular amenazada de España. Dirección General de Medio Natural y Política Forestal (Ministerio de Medio Ambiente, y Medio Rural y Marino), Madrid, 242 pp. |
| *Limonium malacitanum* | Iriondo, J.M., Albert M. J., Gimenez-Benavides, L., Dominguez-Lozano, F. & Escudero, A. [Eds.] (2009): Poblaciones en peligro: viabilidad demográfica de la flora vascular amenazada de España. Dirección General de Medio Natural y Política Forestal (Ministerio de Medio Ambiente, y Medio Rural y Marino), Madrid, 242 pp. |
| *Lindera umbellate* subsp. *membrancea* | Hara, M., Kanno, H., Hirabuki, Y., & Takehara, A. (2004). Population dynamics of four understorey shrub species in beech forest. Journal of Vegetation Science, 15(4), 475–484. Portico. https://doi.org/10.1111/j.1654-1103.2004.tb02286.x |
| *Linum catharticum* | Verkaar, H. J., & Schenkeveld, A. J. (1984). On the ecology of short-lived forbs in chalk grasslands: life-history characteristics. New Phytologist, 98(4), 659–672. https://doi.org/10.1111/j.1469-8137.1984.tb04155.x |
| *Linum flavum* | Münzbergová, Z. (2013). Comparative demography of two co-occurring *Linum* species with different distribution patterns. Plant Biology, 15(6), 963–970. https://doi.org/10.1111/plb.12007 |
| *Linum tenuifolium* | Münzbergová, Z. (2013). Comparative demography of two co-occurring *Linum* species with different distribution patterns. Plant Biology, 15(6), 963–970. https://doi.org/10.1111/plb.12007 |
| *Lithospermum ruderale* | Bricker, M., & Maron, J. (2012). Postdispersal seed predation limits the abundance of a long-lived perennial forb (*Lithospermum ruderale*). Ecology, 93(3), 532–543. https://doi.org/10.1890/11-0948.1 |
| *Lomatium bradshawii* | Kaye, T. N., Pendergrass, K. L., Finley, K., & Kauffman, J. B. (2001). The effect of fire on the population viability of an endangered prairie plant. Ecological Applications, 11(5), 1366–1380. https://doi.org/10.1890/1051-0761(2001)011[1366:teofot]2.0.co;2 |
| *Lomatium cookii* | Kaye, T. N., & Pyke, D. A. (2003). The effect of stochastic technique on estimates of population viability from transition matrix models. Ecology, 84(6), 1464–1476. https://doi.org/10.1890/0012-9658(2003)084[1464:teosto]2.0.co;2 |
| *Lophophora diffusa* | Diaz Segura O. (2013): Dinámica poblacional de *Lophophora diffusa* "peyote" (Cactaceae) en una localidad del Estado de Querétaro. MSc. Thesis, Universidad Autónoma Metropolitana, Mexico. |
| *Lupinus arboreus* | Kauffman, M. J., & Maron, J. L. (2006). Consumers limit the abundance and dynamics of a perennial shrub with a seed bank. The American Naturalist, 168(4), 454–470. https://doi.org/10.1086/507877 |
| *Lupinus lepidus* var. *lobii* | Bishop, J. G. (1996): Demographic and population genetic variation during colonization by the herb *Lupinus lepidus* on Mount St. Helens. PhD Thesis, University of Washington. |
| *Lupinus tidestromii* | Dangremond, E. M., Pardini, E. A., & Knight, T. M. (2010). Apparent competition with an invasive plant hastens the extinction of an endangered lupine. Ecology, 91(8), 2261–2271. https://doi.org/10.1890/09-0418.1 |
| *Magnolia dealbata* | Sánchez‐Velásquez, L. R., & Pineda‐López, M. del R. (2009). Comparative demographic analysis in contrasting environments of Magnolia dealbata: an endangered species from Mexico. Population Ecology, 52(1), 203–210. Portico. https://doi.org/10.1007/s10144-009-0161-5 |
| *Malacothrix indecora* | Levine, J. M., McEachern, A. K., & Cowan, C. (2008). Rainfall effects on rare annual plants. Journal of Ecology, 96(4), 795–806. https://doi.org/10.1111/j.1365-2745.2008.01375.x |
| *Mammillaria hernandezii* | Rodríguez-Ortega C. (2008). Consecuencias demográficas y evolutivas del secuestro de semillas en tres especies del género *Mammillaria*  (Cactaceae). PhD Dissertation, Universidad Autónoma Metropolitana, Mexico. |
| *Mammillaria huitzilopochtli* | Martínez, A. F., Medina, G. I. M., Golubov, J., Montaña, C., & Mandujano, M. C. (2010). Demography of an endangered endemic rupicolous cactus. Plant Ecology, 210(1), 53–66. https://doi.org/10.1007/s11258-010-9737-6 |
| *Mammillaria magnimamma* | Valverde, T., Quijas, S., López-Villavicencio, M., & Castillo, S. (2004). Population dynamics of *Mammillaria magnimamma* Haworth. (Cactaceae) in a lava-field in central Mexico. Plant Ecology (Formerly Vegetatio), 170(2), 167–184. https://doi.org/10.1023/b:vege.0000021662.78634.de |
| *Mammillaria solisioides* | Rodríguez-Ortega C. (2008). Consecuencias demográficas y evolutivas del secuestro de semillas en tres especies del género *Mammillaria*  (Cactaceae). PhD Dissertation, Universidad Autónoma Metropolitana, Mexico. |
| *Manilkara zapota* | Cruz-Rodríguez, J. A., López-Mata, L., & Valverde, T. (2009). A comparison of traditional elasticity and variance-standardized perturbation analyses: a case study with the tropical tree species *Manilkara zapota* (Sapotaceae). Journal of Tropical Ecology, 25(2), 135–146. https://doi.org/10.1017/s0266467408005713 |
| *Miconia albicans* | Hoffmann, W. A. (1999).Fire and population dynamics of woody plants in a neotropical savanna: matrix model projections. Ecology, 80(4), 1354–1369. https://doi.org/10.1890/0012-9658(1999)080[1354:fapdow]2.0.co;2 |
| *Mimulus cardinalis* | Angert, A. L. (2006). Demography of central and marginal populations of monkeyflowers (*Mimulus cardinalisandm*. Lewisii). Ecology, 87(8), 2014–2025. https://doi.org/10.1890/0012-9658(2006)87[2014:docamp]2.0.co;2 |
| *Mimulus lewisii* | Angert, A. L. (2006). Demography of central and marginal populations of monkeyflowers (*Mimulus cardinalisandm*. Lewisii). Ecology, 87(8), 2014–2025. https://doi.org/10.1890/0012-9658(2006)87[2014:docamp]2.0.co;2 |
| *Miscanthus giganteus* | Matlaga, D. P., & Davis, A. S. (2013). Minimizing invasive potential of *Miscanthus × giganteus* grown for bioenergy: identifying demographic thresholds for population growth and spread. Journal of Applied Ecology, 50(2), 479–487. Portico. https://doi.org/10.1111/1365-2664.12057 |
| *Mitrocereus fulviceps* | Vite González, F. & J. Zavala Hurtado, J. A. (1998). Estatus ecológicos de *Mammillaria pectinifera* Weber y *Pachycereus fulviceps* Weber en el Valle de Zapotitlán, Puebla. Universidad Autónoma Metropolitana-Iztapalapa. División de Ciencias Biológicas y de la Salud. Informe final SNIB- CONABIO proyecto No. G022. México D. F. |
| *Molinia caerulea* | Jacquemyn, H., Brys, R., & Neubert, M. G. (2005). Fire increases invasive spread of *Molinia caerulea* mainly through changes in demographic parameters. Ecological Applications, 15(6), 2097–2108. https://doi.org/10.1890/04-1762 |
| *Mulinum spinosum* | Cipriotti, P. A., & Aguiar, M. R. (2011). Direct and indirect effects of grazing constrain shrub encroachment in semi-arid Patagonian steppes. Applied Vegetation Science, 15(1), 35–47. https://doi.org/10.1111/j.1654-109x.2011.01138.x |
| *Myosotis ramosissima* | Dostál, P. (2007). Population dynamics of annuals in perennial grassland controlled by ants and environmental stochasticity. Journal of Vegetation Science, 18(1), 91–102. Portico. https://doi.org/10.1111/j.1654-1103.2007.tb02519.x |
| *Narcissus pseudonarcissus* | Barkham, J. P. (1980). Population dynamics of the wild daffodil (Narcissus pseudonarcissus): I. Clonal growth, seed reproduction, mortality and the effects of density. The Journal of Ecology, 68(2), 607. https://doi.org/10.2307/2259425 |
| *Neobuxbaumia macrocephala* | Esparza‐Olguín, L., Valverde, T., & Mandujano, M. C. (2005). Comparative demographic analysis of three *Neobuxbaumia species* (Cactaceae) with differing degree of rarity. Population Ecology, 47(3), 229–245. Portico. https://doi.org/10.1007/s10144-005-0230-3 |
| *Neobuxbaumia mezcalaensis* | Esparza‐Olguín, L., Valverde, T., & Mandujano, M. C. (2005). Comparative demographic analysis of three *Neobuxbaumia species* (Cactaceae) with differing degree of rarity. Population Ecology, 47(3), 229–245. Portico. https://doi.org/10.1007/s10144-005-0230-3 |
| *Neobuxbaumia polylopha* | Arroyo-Cosultchi, G., Golubov, J., & Mandujano, M. C. (2016). Pulse seedling recruitment on the population dynamics of a columnar cactus: Effect of an extreme rainfall event. Acta Oecologica, 71, 52–60. https://doi.org/10.1016/j.actao.2016.01.006 |
| *Neodypsis decaryi* | Ratsirarson, J., Silander, J. A., & Richard, A. F. (1996). Conservation and management of a threatened Madagascar palm species, Neodypsis decaryi, Jumelle. Conservation Biology, 10(1), 40–52. https://doi.org/10.1046/j.1523-1739.1996.10010040.x |
| *Oenothera deltoides subsp. howellii* | Thompson, D. M. (2005). Matrix models as a tool for understanding invasive plant and native plant interactions. Conservation Biology, 19(3), 917–928. https://doi.org/10.1111/j.1523-1739.2005.004108.x |
| *Orchis purpurea* | Jacquemyn, H., Brys, R., & Jongejans, E. (2010). Seed limitation restricts population growth in shaded populations of a perennial woodland orchid. Ecology, 91(1), 119–129. https://doi.org/10.1890/08-2321.1 |
| *Pachycereus pecten-aboriginum* | Morales-Romero, D., Godínez-Álvarez, H., Campo-Alves, J., & Molina-Freaner, F. (2012). Effects of land conversion on the regeneration of *Pachycereus pecten-aboriginum* and its consequences on the population dynamics in northwestern Mexico. Journal of Arid Environments, 77, 123–129. https://doi.org/10.1016/j.jaridenv.2011.09.005 |
| *Paeonia officinalis* | Andrieu, E., Fréville, H., Besnard, A., Vaudey, V., Gauthier, P., Thompson, J. D., & Debussche, M. (2012). Forest‐cutting rapidly improves the demographic status of *Paeonia officinalis*, a species threatened by forest closure. Population Ecology, 55(1), 147–158. Portico. https://doi.org/10.1007/s10144-012-0346-1 |
| *Panax quinquefolius* | Farrington, S. J., Muzika, R.-M., Drees, D., & Knight, T. M.(2009).Interactive effects of harvest and deer herbivory on the population dynamics of American ginseng. Conservation Biology, 23(3), 719–728. https://doi.org/10.1111/j.1523-1739.2008.01136.x |
| *Parkinsonia aculeata* | Raghu, S., Wilson, J. R., & Dhileepan, K. (2006). Refining the process of agent selection through understanding plant demography and plant response to herbivory. Australian Journal of Entomology, 45(4), 308–316. https://doi.org/10.1111/j.1440-6055.2006.00556.x |
| *Paronychia pulvinata* | Forbis, T. A., & Doak, D. F. (2004). Seedling establishment and life history trade‐offs in alpine plants. American Journal of Botany, 91(7), 1147–1153. Portico. https://doi.org/10.3732/ajb.91.7.1147 |
| *Pedicularis furbishiae* | Menges, E. S. (1990). Population viability analysis for an endangered plant. Conservation Biology, 4(1), 52–62. https://doi.org/10.1111/j.1523-1739.1990.tb00267.x |
| *Periandra mediterranea* | Hoffmann, W. A., & Solbrig, O. T. (2003). The role of topkill in the differential response of savanna woody species to fire. Forest Ecology and Management, 180(1–3), 273–286. https://doi.org/10.1016/s0378-1127(02)00566-2 |
| *Persoonia bargoensis* | McKenna D. J. (2007). Demographic and ecological indicators of rarity in a suite of obligate-seeding *Persoonia* (Proteaceae) shrubs. PhD Thesis, University of Wollongong. |
| *Persoonia glaucescens* | McKenna D. J. (2007). Demographic and ecological indicators of rarity in a suite of obligate-seeding *Persoonia* (Proteaceae) shrubs. PhD Thesis, University of Wollongong. |
| *Petrocoptis pseudoviscosa* | Garcı́a, M. B., Guzmán, D., & Goñi, D. (2002). An evaluation of the status of five threatened plant species in the Pyrenees. Biological Conservation, 103(2), 151–161. https://doi.org/10.1016/s0006-3207(01)00113-6 |
| *Petrophile pulchella* | Bradstock, R. A., & O’Connell, M. A. (1988). Demography of woody plants in relation to fire: *Banksia ericifolia* L.f. and *Petrophile pulchella* (Schrad) R.Br. Austral Ecology, 13(4), 505–518. https://doi.org/10.1111/j.1442-9993.1988.tb00999.x |
| *Phacelia insularis* var. *insularis* | Levine, J. M., McEachern, A. K., & Cowan, C. (2008). Rainfall effects on rare annual plants. Journal of Ecology, 96(4), 795–806. https://doi.org/10.1111/j.1365-2745.2008.01375.x |
| *Phaseolus lunatus* | Degreef, J., Baudoin, J.-P., & Rocha, O. J. (1997). Case studies on breeding systems and its consequences for germplasm conservation. Genetic Resources and Crop Evolution, 44(5), 429–438. https://doi.org/10.1023/a:1008623521755 |
| *Phyllanthus emblica* | Ellis, M. M., Williams, J. L., Lesica, P., Bell, T. J., Bierzychudek, P., Bowles, M., Crone, E. E., Doak, D. F., Ehrlén, J., Ellis-Adam, A., McEachern, K., Ganesan, R., Latham, P., Luijten, S., Kaye, T. N., Knight, T. M., Menges, E. S., Morris, W. F., Nijs, H. den, … Weekley, C. W. (2012). Matrix population models from 20 studies of perennial plant populations. Ecology, 93(4), 951–951. Portico. https://doi.org/10.1890/11-1052.1 |
| *Phyllanthus indofischeri* | Ticktin, T., Ganesan, R., Paramesha, M., & Setty, S. (2012). Disentangling the effects of multiple anthropogenic drivers on the decline of two tropical dry forest trees. Journal of Applied Ecology, 49(4), 774–784. https://doi.org/10.1111/j.1365-2664.2012.02156.x |
| *Phytelephas seemannii* | Bernal, R. (1998). Demography of the vegetable ivory palm *Phytelephas seemannii* in Colombia, and the impact of seed harvesting. Journal of Applied Ecology, 35(1), 64–74. Portico. https://doi.org/10.1046/j.1365-2664.1998.00280.x |
| *Picris hieracioides* | Klemow, K. M., & Raynal, D. J. (1985). Demography of two facultative biennial plant species in an unproductive habitat. The Journal of Ecology, 73(1), 147. https://doi.org/10.2307/2259775 |
| *Pimpinella saxifraga* | Auestad, I., Rydgren, K., Jongejans, E., & Kroon, H. de. (2010). *Pimpinella saxifraga* is maintained in road verges by mosaic management. Biological Conservation, 143(4), 899–907. https://doi.org/10.1016/j.biocon.2009.12.037 |
| *Pinguicula alpina* | Svensson, B. M., Carlsson, B. A., Karlsson, P. S., & Nordell, K. O. (1993). Comparative long-term demography of three species of *Pinguicula*. The Journal of Ecology, 81(4), 635. https://doi.org/10.2307/2261662 |
| *Pinguicula villosa* | Svensson, B. M., Carlsson, B. A., Karlsson, P. S., & Nordell, K. O. (1993). Comparative long-term demography of three species of *Pinguicula*. The Journal of Ecology, 81(4), 635. https://doi.org/10.2307/2261662 |
| *Pinus albicaulis* | Ettl, G., & N. Cottone (2004). Whitebark pine (*Pinus albicaulis*) in Mt. Rainier National Park: response to blister rust infection. Pages 36–47 in H. Akc akaya, M. Burgman, O.Kindvall, C. Wood, P. Sjogren-Gulve, J. Hatfield, and M.McCarthy (Eds.). Species conservation and management. Oxford University Press, New York, New York, USA |
| *Pinus kwangtungensis* | Chien, P. D., Zuidema, P. A., & Nghia, N. H. (2008). Conservation prospects for threatened Vietnamese tree species: results from a demographic study. Population Ecology, 50(2), 227–237. Portico. https://doi.org/10.1007/s10144-008-0079-3 |
| *Pinus lambertiana* | Van Mantgem, P. J., & Stephenson, N. L. (2005). The accuracy of matrix population model projections for coniferous trees in the Sierra Nevada, California. Journal of Ecology, 93(4), 737–747. Portico. https://doi.org/10.1111/j.1365-2745.2005.01007.x |
| *Pinus maximartinezii* | López-Mata, L. (2013). The impact of seed extraction on the population dynamics of *Pinus maximartinezii*. Acta Oecologica, 49, 39–44. https://doi.org/10.1016/j.actao.2013.02.010 |
| *Pinus nigra* subsp. *lauricio* | Buckley, Y. M., Brockerhoff, E., Langer, L., Ledgard, N., North, H., & Rees, M.(2005). Slowing down a pine invasion despite uncertainty in demography and dispersal. Journal of Applied Ecology, 42(6), 1020–1030. https://doi.org/10.1111/j.1365-2664.2005.01100.x |
| *Pinus strobus* | Münzbergová, Z., Hadincová, V., Wild, J., & Kindlmannová, J. (2013). Variability in the contribution of different life stages to population growth as a key factor in the invasion success of *Pinus strobus*. PLoS ONE, 8(2), e56953. https://doi.org/10.1371/journal.pone.0056953 |
| *Pinus sylvestris* | Usher, M. B. (1966). A matrix approach to the management of renewable resources, with special reference to selection forests. The Journal of Applied Ecology, 3(2), 355. https://doi.org/10.2307/2401258 |
| *Plantago coronopus* | Villellas, J., Ehrlén, J., Olesen, J. M., Braza, R., & García, M. B. (2012). Plant performance in central and northern peripheral populations of the widespread *Plantago coronopus*. Ecography, 36(2), 136–145. https://doi.org/10.1111/j.1600-0587.2012.07425.x |
| *Plantago media* | Eriksson, Å., & Eriksson, O. (2000). Population dynamics of the perennial *Plantago media* in semi-natural grasslands. Journal of Vegetation Science, 11(2), 245–252. Portico. https://doi.org/10.2307/3236803 |
| *Polemonium van-bruntiae* | Hill Bermingham, L. (2010). Deer herbivory and habitat type influence long-term population dynamics of a rare wetland plant. Plant Ecology, 210(2), 359–378. https://doi.org/10.1007/s11258-010-9762-5 |
| *Polygonella basiramia* | Maliakal Witt, S. (2004): Microhabitat distribution and demography of two Florida scrub endemic plants with comparisons to their habitat-generalist congeners. PhD Thesis, Louisiana State Eniversity, Louisiana. |
| *Potentilla anserina* | Eriksson, O. (1988). Ramet behaviour and population growth in the clonal herb *Potentilla anserina*. The Journal of Ecology, 76(2), 522. https://doi.org/10.2307/2260610 |
| *Primula elatior* | Jacquemyn, H., & Brys, R. (2008). Effects of stand age on the demography of a temperate forest herb in post-agricultural forests. Ecology, 89(12), 3480–3489. https://doi.org/10.1890/07-1908.1 |
| *Primula veris* | Ehrlén, J., Syrjänen, K., Leimu, R., Begoña Garcia, M., & Lehtilä, K. (2005). Land use and population growth of *Primula veris*: an experimental demographic approach. Journal of Applied Ecology, 42(2), 317–326. https://doi.org/10.1111/j.1365-2664.2005.01015.x |
| *Primula vulgaris* | Valverde, T., & Silvertown, J. (1998). Variation in the demography of a woodland understorey herb (*Primula vulgaris*) along the forest regeneration cycle: projection matrix analysis. Journal of Ecology, 86(4), 545–562. Portico. https://doi.org/10.1046/j.1365-2745.1998.00280.x |
| *Prosopis glandulosa* | Golubov, J., Mandujano, M. D. C., Franco, M., Montana, C., Eguiarte, L. E., & Lopez-Portillo, J. (1999). Demography of the invasive woody perennial *Prosopis glandulosa* (honey mesquite). Journal of Ecology, 87(6), 955–962. https://doi.org/10.1046/j.1365-2745.1999.00420.x |
| *Prosopis laevigata* | Bernal R. (2010). Comportamiento demográfico y la dinámica espacio-temporal de la planta epífita *Tillandsia recurvata* L. (Bromeliaceae). PhD thesis. Universidad Nacional Autónoma de Mexico. |
| *Prunus africana* | Stewart, K. M. 2001. The commercial bark harvest of the African cherry (*Prunus africana*) on Mount Oku, Cameroon: effects on traditional uses and population dynamics. Ph.D. dissertation, Florida International University, Miami, FL. |
| *Prunus serotina* | Sebert-Cuvillier, E., Paccaut, F., Chabrerie, O., Endels, P., Goubet, O., & Decocq, G. (2007). Local population dynamics of an invasive tree species with a complex life-history cycle: A stochastic matrix model. Ecological Modelling, 201(2), 127–143. https://doi.org/10.1016/j.ecolmodel.2006.09.005 |
| *Pseudophoenix sargentii* | Durán, R. & R. Franco. 1992. Estudio demográfico de *Pseudophoenix sargentii*. Bulletin de l'Institut Français d'Études Andines 21: 609-621. |
| *Purshia subintegra* | Maschinski, J., Baggs, J. E., Quintana-Ascencio, P. F., & Menges, E. S. (2006). Using population viability analysis to predict the effects of climate change on the extinction risk of an endangered limestone endemic shrub, arizona cliffrose. Conservation Biology, 20(1), 218–228. https://doi.org/10.1111/j.1523-1739.2006.00272.x |
| *Quercus crispula* | Hiura, T., & Fujiwara, K. (1999). Density‐dependence and co‐existence of conifer and broad‐leaved trees in a Japanese northern mixed forest. Journal of Vegetation Science, 10(6), 843–850. Portico. https://doi.org/10.2307/3237309 |
| *Quercus rugosa* | Bonfi, C. (2006): Regeneration and population dynamics of *Quercus rugosa* at the Ajusco Volcano, Mexico. In: Kappelle, M. (eds) Ecology and Conservation of Neotropical Montane Oak Forests. Ecological Studies, vol 185. Springer, Berlin, Heidelberg. https://doi.org/10.1007/3-540-28909-7_12 |
| *Ranunculus acris* | Sarukhan, J., & Harper, J. L. (1973). Studies on plant demography: *Ranunculus repens* L., *R. bulbosus* L. and *R. acris* L.: I. Population flux and survivorship. The Journal of Ecology, 61(3), 675. https://doi.org/10.2307/2258643 |
| *Rhizophora mangle* | López-Hoffman, L., Ackerly, D. D., Anten, N. P. R., Denoyer, J. L., & Martinez-Ramos, M. (2007). Gap-dependence in mangrove life-history strategies: a consideration of the entire life cycle and patch dynamics. Journal of Ecology, 95(6), 1222–1233. https://doi.org/10.1111/j.1365-2745.2007.01298.x |
| *Rhododendron ponticum* | Salguero-Gomez R. 2004. Markov Chains applied to *Rhododendron ponticum* L.: ecological terminator in Great Britain ecologically terminated in Spain? MSc thesis. Kingston University, London. |
| *Rubus discolor* | Lambrecht-McDowell, S. C., & Radosevich, S. R. (2005). Population demographics and trade-offs to reproduction of an invasive and noninvasive species of *Rubus*. Biological Invasions, 7(2), 281–295. https://doi.org/10.1007/s10530-004-0870-9 |
| *Rubus ursinus* | Lambrecht-McDowell, S. C., & Radosevich, S. R. (2005). Population demographics and trade-offs to reproduction of an invasive and noninvasive species of *Rubus*. Biological Invasions, 7(2), 281–295. https://doi.org/10.1007/s10530-004-0870-9 |
| *Sabal minor* | Ramp, P.F. (1989) Natural history of *Sabal minor*: Demography, population genetics, and reproductive biology. Ph.D. dissertation, Tulane University, New Orleans, Louisiana, 211 pp. |
| *Salsola australis* | Borger, C. P. D., Scott, J. K., Renton, M., Walsh, M., & Powles, S. B. (2009). Assessment of management options for *Salsola australis* in south-west Australia by transition matrix modelling. Weed Research, 49(4), 400–408. https://doi.org/10.1111/j.1365-3180.2009.00703.x |
| *Sanicula europaea* | Gustafsson, C., & Ehrlén, J. (2003). Effects of intraspecific and interspecific density on the demography of a perennial herb, *Sanicula europaea*. Oikos, 100(2), 317–324. Portico. https://doi.org/10.1034/j.1600-0706.2003.11493.x |
| *Sarcocapnos baetica* | Salinas, M. J., Suárez, V., & Blanca, G. (2002). Demographic structure of three species of *Sarcocapnos* (Fumariaceae) as a basis for their conservation. Canadian Journal of Botany, 80(4), 360–369. https://doi.org/10.1139/b02-013 |
| *Sarcocapnos enneaphylla* | Salinas, M. J., Suárez, V., & Blanca, G. (2002). Demographic structure of three species of *Sarcocapnos* (Fumariaceae) as a basis for their conservation. Canadian Journal of Botany, 80(4), 360–369. https://doi.org/10.1139/b02-013 |
| *Sarcocapnos pulcherrima* | Salinas, M. J., Suárez, V., & Blanca, G. (2002). Demographic structure of three species of *Sarcocapnos* (Fumariaceae) as a basis for their conservation. Canadian Journal of Botany, 80(4), 360–369. https://doi.org/10.1139/b02-013 |
| *Sarracenia alata* | Brewer, J. S. (2001). A demographic analysis of fire-stimulated seedling establishment of *Sarracenia alata* (Sarraceniaceae). American Journal of Botany, 88(7), 1250–1257. Portico. https://doi.org/10.2307/3558336 |
| *Saussurea medusa* | Law, W., Salick, J., & Knight, T. M. (2010). The effects of pollen limitation on population dynamics of snow lotus (*Saussurea medusa* and *S. laniceps*, Asteraceae): Threatened Tibetan medicinal plants of the eastern Himalayas. Plant Ecology, 210(2), 343–357. https://doi.org/10.1007/s11258-010-9761-6 |
| *Saxifraga tridactylites* | Dostál, P. (2007). Population dynamics of annuals in perennial grassland controlled by ants and environmental stochasticity. Journal of Vegetation Science, 18(1), 91–102. Portico. https://doi.org/10.1111/j.1654-1103.2007.tb02519.x |
| *Scabiosa columbaria* | Verkaar, H. J., & Schenkeveld, A. J. (1984). On the ecology of short-lived forbs in chalk grasslands: life-history characteristics. New Phytologist, 98(4), 659–672. https://doi.org/10.1111/j.1469-8137.1984.tb04155.x |
| *Scorzonera hispanica* | Münzbergová, Z. (2006). Effect of population size on the prospect of species survival. Folia Geobotanica, 41(2), 137–150. https://doi.org/10.1007/bf02806475 |
| *Senecio filaginoides* | Cipriotti, P. A., & Aguiar, M. R. (2011). Direct and indirect effects of grazing constrain shrub encroachment in semi-arid Patagonian steppes. Applied Vegetation Science, 15(1), 35–47. https://doi.org/10.1111/j.1654-109x.2011.01138.x |
| *Shorea acuminata* | Yamada, T., Yamada, Y., Okuda, T., & Fletcher, C. (2012). Soil-related variations in the population dynamics of six dipterocarp tree species with strong habitat preferences. Oecologia, 172(3), 713–724. https://doi.org/10.1007/s00442-012-2529-z |
| *Shorea bracteolata* | Yamada, T., Yamada, Y., Okuda, T., & Fletcher, C. (2012). Soil-related variations in the population dynamics of six dipterocarp tree species with strong habitat preferences. Oecologia, 172(3), 713–724. https://doi.org/10.1007/s00442-012-2529-z |
| *Silene acaulis* | Morris, W. F., & Doak, D. F. (1998). Life history of the long‐lived gynodioecious cushion plant *Silene acaulis* (Caryophyllaceae), inferred from size‐based population projection matrices. American Journal of Botany, 85(6), 784–793. Portico. https://doi.org/10.2307/2446413 |
| *Silene spaldingii* | Lesica, P., & Crone, E. E. (2007). Causes and consequences of prolonged dormancy for an iteroparous geophyte, *Silene spaldingii*. Journal of Ecology, 95(6), 1360–1369. https://doi.org/10.1111/j.1365-2745.2007.01291.x |
| *Sonchus pustulatus* | Silva, J. L., Mejías, J. A., & García, M. B. (2015). Demographic vulnerability in cliff-dwelling *Sonchus* species endemic to the western Mediterranean. Basic and Applied Ecology, 16(4), 316–324. https://doi.org/10.1016/j.baae.2015.02.009 |
| *Stenocereus eruca* | Clark-Tapia, R., Mandujano, M. C., Valverde, T., Mendoza, A., & Molina-Freaner, F. (2005). How important is clonal recruitment for population maintenance in rare plant species?: the case of the narrow endemic cactus, *Stenocereus eruca*, in Baja California, México. Biological Conservation, 124(1), 123–132. https://doi.org/10.1016/J.BIOCON.2005.01.019 |
| *Stryphnodendron excelsum* | Hartshorn, G. S. 1972. The ecological life history and population dynamics of Pen-taclethra macroloba, a tropical wet forest dominant and *Stryphnodendron excel-sum*, an occasional associate. Ph.D. Thesis, University Washington, Seattle. |
| *Succisa pratensis* | Milden, M. (2005): Local and regional dynamics of Succisa pratensis. PhD Thesis, University of Stockholm. |
| *Syzygium jambos* | Brown, K. A., Spector, S., & Wu, W. (2008). Multi-scale analysis of species introductions: combining landscape and demographic models to improve management decisions about non-native species. Journal of Applied Ecology, 45(6), 1639–1648. https://doi.org/10.1111/j.1365-2664.2008.01550.x |
| *Taxus floridana* | Kwit, C., Horvitz, C. C., & Platt, W. J. (2004). Conserving slow-growing, long-lived tree species: input from the demography of a rare understory conifer, *Taxus floridana*. Conservation Biology, 18(2), 432–443. https://doi.org/10.1111/j.1523-1739.2004.00567.x |
| *Thymus webbianus* | Iriondo, J.M., Albert M. J., Gimenez-Benavides, L., Dominguez-Lozano, F. & Escudero, A. [Eds.] (2009): Poblaciones en peligro: viabilidad demográfica de la flora vascular amenazada de España. Dirección General de Medio Natural y Política Forestal (Ministerio de Medio Ambiente, y Medio Rural y Marino), Madrid, 242 pp. |
| *Tillandsia macdougallii* | Mondragón Chaparro, D., & Ticktin, T. (2011). Demographic effects of harvesting epiphytic bromeliads and an alternative approach to collection. Conservation Biology, 25(4), 797–807. https://doi.org/10.1111/j.1523-1739.2011.01691.x |
| *Tillandsia multicaulis* | Winkler, M., Hülber, K., & Hietz, P. (2007). Population dynamics of epiphytic bromeliads: Life strategies and the role of host branches. Basic and Applied Ecology, 8(2), 183–196. https://doi.org/10.1016/j.baae.2006.05.003 |
| *Tillandsia punctulata* | Toledo-Aceves, T., Hernández-Apolinar, M., & Valverde, T. (2014). Potential impact of harvesting on the population dynamics of two epiphytic bromeliads. Acta Oecologica, 59, 52–61. https://doi.org/10.1016/j.actao.2014.05.009 |
| *Tillandsia violacea* | Mondragón Chaparro, D., & Ticktin, T. (2011). Demographic effects of harvesting epiphytic bromeliads and an alternative approach to collection. Conservation Biology, 25(4), 797–807. https://doi.org/10.1111/j.1523-1739.2011.01691.x |
| *Trillium grandiflorum* | Knight, T. M. (2003). Effects of herbivory and its timing across populations of *Trillium grandiflorum* (Liliaceae). American Journal of Botany, 90(8), 1207–1214. Portico. https://doi.org/10.3732/ajb.90.8.1207 |
| *Trollius europaeus* | Lemke, T., & Salguero-Gómez, R. (2015). Land use heterogeneity causes variation in demographic viability of a bioindicator of species-richness in protected fen grasslands. Population Ecology, 58(1), 165–178. https://doi.org/10.1007/s10144-015-0519-9 |
| *Trollius laxus* | Scanga, S. E., & Leopold, D. J. (2012). Managing wetland plant populations: Lessons learned in Europe may apply to North American fens. Biological Conservation, 148(1), 69–78. https://doi.org/10.1016/j.biocon.2012.01.061 |
| *Tsuga canadensis* | Lamar, W. R., & McGraw, J. B. (2005). Evaluating the use of remotely sensed data in matrix population modeling for eastern hemlock (*Tsuga canadensis* L.). Forest Ecology and Management, 212(1–3), 50–64. https://doi.org/10.1016/j.foreco.2005.02.056 |
| *Veronica arvensis* | Dostál, P. (2007). Population dynamics of annuals in perennial grassland controlled by ants and environmental stochasticity. Journal of Vegetation Science, 18(1), 91–102. Portico. https://doi.org/10.1111/j.1654-1103.2007.tb02519.x |
| *Verticordia fimbrilepis* subsp*. fimbrilepis* | Yates, C. J., & Ladd, P. G. (2010). Using population viability analysis to predict the effect of fire on the extinction risk of an endangered shrub *Verticordia fimbrilepis* subsp. *fimbrilepis* in a fragmented landscape. Plant Ecology, 211(2), 305–319. https://doi.org/10.1007/s11258-010-9791-0 |
| *Viola fimbriatula* | Solbrig, O. T., Sarandon, R., & Bossert, W. (1988). A density-dependent growth model of a perennial herb, *Viola fimbriatula*. The American Naturalist, 131(3), 385–400. https://doi.org/10.1086/284796 |
| *Vochysia ferruginea* | Boucher, D. H., & Mallona, M. A. (1997). Recovery of the rain forest tree *Vochysia ferruginea* over 5 years following Hurricane Joan in Nicaragua: a preliminary population projection matrix. Forest Ecology and Management, 91(2–3), 195–204. https://doi.org/10.1016/s0378-1127(96)03890-x |
| *Zamia inermis* | Octavio-Aguilar, P., Rivera-Fernández, A., Iglesias-Andreu, L. G., Vovides, P. A., & de Cáceres-González, F. F. N. (2017). Extinction risk of *Zamia inermis*: a demographic study in its single natural population. Biodiversity and Conservation, 26(4), 787–800. https://doi.org/10.1007/s10531-016-1270-z |
| *Zea diploperennis* | Sanchez-Velasquez, L. R., Ezcurra, E., Martinez-Ramos, M., Alvarez-Buylla, E., & Lorente, R. (2002). Population dynamics of *Zea diploperennis*, an endangered perennial herb: effect of slash and burn practice. Journal of Ecology, 90(4), 684–692. https://doi.org/10.1046/j.1365-2745.2002.00702.x |
