# Supplementary material for: Assessing limits of sustainable seed harvest in wild plant populations
Source: Conserv Biol. 2025 May 31;39(5):e70075. doi: 10.1111/cobi.70075 (PMC12451500; doi:10.1111/cobi.70075)
Supplement: Supplementary file 2 — Supporting Information [file COBI-39-e70075-s002.docx]

Appendix S2

Materials and Methods, Supplementary results

**Assessing limits of sustainable seed harvest in wild plant populations**

Anna Bucharova, Oliver Bossdorf, J.F. Scheepens, Roberto Salguero-Gómez

Materials and Methods, Supplementary results

To quantify the effect of seed harvesting on wild plant populations, we used matrix population models (Caswell 2001). We first tested the impacts of seed harvesting by simulating the regulatory recommendations on seed harvesting in the wild of three regions where such regulations are in place (Australia, Germany and USA). Second, we calculated the population vulnerability to seed harvesting for each of the 280 plant species examined. Third, we related those effects to plant key life history traits (*i.e.* defining characteristics of their life cycles; e.g. generation time, age at maturity). In the fourth step, we used the life history traits that explained most of the vulnerability of natural populations to seed harvesting to formulate biologically-sound management recommendations. The ultimate goal of these recommendations is to introduce a threshold to seed harvesting so that (i) the population size does not decline more than by 50% over 30 years of consecutive (*i.e.* annual) seed harvest and (ii) the population may still have a 95% probability of persistence. All calculations and statistics were performed in R (R Development Core Team 2015), and the reproducible, commented scripts are found as *Auxiliary material and will be available at Zonedo upon acceptance*.

# Matrix population models

## General introduction

Matrix population models (MPMs, hereafter) are a widely used tool for investigating population dynamics (Caswell 2001). Briefly, an MPM describes the life cycle of an organism in terms of age, size and/or developmental stages along its life cycle and the transitions between stages, usually from one year to the next, as well as the sexual and clonal per-capita contributions to the population by individuals in each of those stages (Figure S1). One of the many applications of MPMs is to project the dynamics of a population through time (Caswell 2001), whereby a long-term population growth rate can be estimated (Figure S1). Importantly here, MPMs can also be used to calculate a wide range of population characteristics such as life history traits (Salguero-Gómez et al. 2016), extinction probability (Morris & Doak 2002), and the effects of different hypothetical events (such as seed harvesting) on the long-term viability of a population (de Kroon et al. 1986, 2000).

In this study, we used MPMs to simulate seed harvesting as reduction of the per-capita contribution(s) describing seed production (Figure S1). We did so by simulating the harvesting of newly produced seeds while keeping all other demographic processes unaltered. The resulting MPM thus describes the population dynamics in a year where seed harvesting took place.


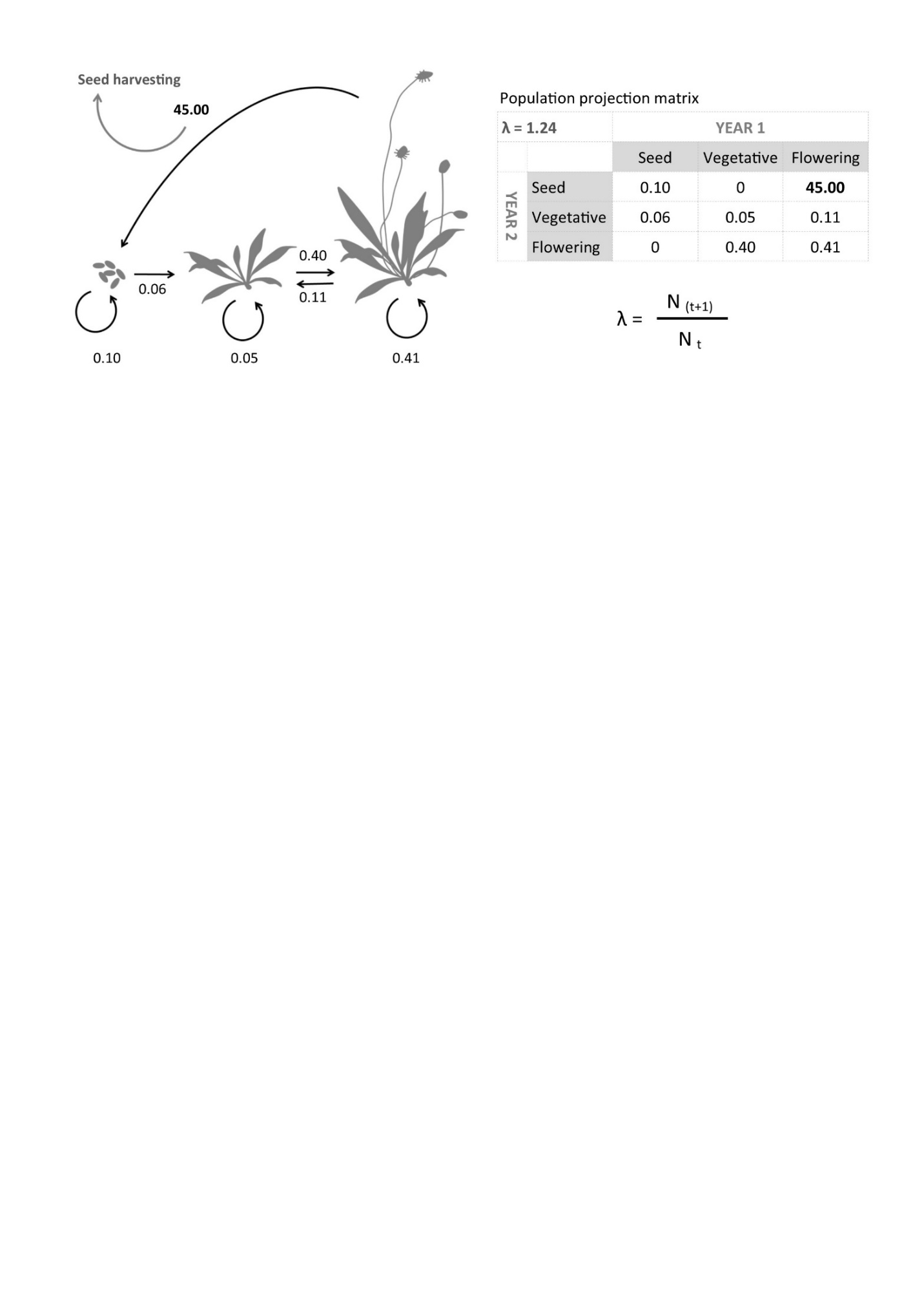


Figure S1: Life cycle of a hypothetical plant species with three stages (seedbank, juvenile, and adult) and its corresponding matrix population model (MPM), with *λ* indicating its long-term population growth rate, which is a function of population size (*N*) between two time-points *t* and *t*+1. Seed harvesting in this study was simulated by manipulating the transitions that describe generative reproduction.

## COMPADRE database

We used data stored in THE COMPADRE Plant Matrix Database (version 5.0.0.), last accessed 25.8.2019 (“COMPADRE Plant Matrix Database (2019). Available from: https://www.compadre-db.org [25.8.2019, Version 5.0.0]” 2019). In this version, COMPADRE contains 9121 MPMs from 647 published works describing life cycles of 760 plant species, ranging from algae to trees worldwide. MPMs in the database are accompanied by extensive metadata including the continent where the study was carried out, whether it was carried out in captivity or in the wild, and standardized information about each life cycle stage in three categories: propagules, individuals photosynthetically active, and individuals in vegetative dormancy. In the vast majority of MPMs in COMPADRE, the full MPM ***A*** is divided into three submatrices (Salguero-Gómez et al. 2016): ***U*** includes demographic processes that depend on survival of individuals alive at the beginning of the census (i.e., progressive growth, stasis, retrogressive growth, seed bank persistence, and vegetative dormancy), ***F*** includes sexual reproduction (e.g. production of seeds and juveniles), and ***C*** includes clonal reproduction (*i.e.* vegetative reproduction through ramets), such that

***A*** = ***U*** + ***F*** + ***C*** *eq. 1*

## Selection of the MPMs

We selected species and MPMs from COMPADRE based on the following criteria to allow for inter-specific comparisons to answer our questions:

- Only angiosperms and gymnosperms, since the ultimate goal of this study is to simulate the effect of seed harvesting on seed-producing plants.
- MPMs parameterised from field data from wild populations and under unmanipulated conditions, because the aim of this study is to understand the effect of seed harvest on natural, wild populations.
- MPMs for which the sexual reproduction component had been quantified explicitly, and separated from other processes in order to allow us to accurately perturb sexual reproduction (seed production; see below).
- MPMs that are irreducible, ergodic, and primitive, so the dominant eigenvalue (population growth rate) and other key properties could be calculated (Caswell 2001).
- When multiple studies per species were available (n = 235 species), we selected the single study per species that:
  - - documented a seed bank, because inclusion of this transition in MPMs is vital to correct estimation of life history traits (Nguyen et al. 2019)
    - contained the highest number of individual MPMs (*i.e.*, from more populations or more years, see SM section 1.4) to use the most representative demographic information for the target species.

These selection criteria resulted in 467 MPMs from 467 plant species. Next, we checked the reliability of incorporating a seed bank in them or not. While survival of seeds in the seed bank is well documented in many demographic studies (Bucharová et al. 2012), between 42.9% and 47.3% of studies using MPMs in plant species unjustifiably exclude seed banks (8), thus assigning seedlings in year *t* to reproductive plants in *t*-1 (e.g. (Menges 1990)). However, this assumption is only correct in species with a transient seed bank, i.e. seeds survive in the soil less than one year and thus, do not form a permanent soil seed bank (Nguyen et al. 2019). For those studies in our list where seed banks were not explicitly considered in their MPMs, we verified whether the species indeed have only a transient seed bank or not. We did so by carefully examining the original source of the MPM(s). If the source did not mention a seed bank, we further searched in the TRY database (Kattge et al. 2011) for its potential existence. Consequently, we excluded 169 species where seed banks were unjustifiably excluded from their MPMs.

In twelve species, the simulated seed harvesting (SM section 2) did not cause any changes of population sizes, which suggests that generative reproduction was not correctly incorporated in these MPMs. We excluded these species from the further analysis.

This final selection criterion resulted in a dataset of 280 species (each with a representative MPM) from 83 plant families. This is the final set of species and data that were used for the simulations described below (Appendix S1).

## Mean MPMs vs individual MPMs

For the majority of studies in COMPADRE, MPMs are available for several annual transitions and populations. This was also the case in our final dataset. For all calculations, except in the case of stochastic simulations (Section 8), we used a single *mean* MPM per species across all years and populations of demographic data available for that species. This mean MPM was calculated as the element-by-element arithmetic mean of the aforementioned MPMs, or pooled directly (e.g. weighted mean by sample size) from the individual-level data when provided by the author in the publication or through personal communications with the COMPADRE team.

For the stochastic simulations we used *individual* MPMs, which represented the population dynamics during a given annual transition and at a given population. We only used species that were represented in the database by at least three individual MPMs (Section 8), resulting in 1578 individual MPMs from across 108 plant species in our dataset.

# Simulating seed harvesting

We used the selected MPMs to simulate the impact of seed harvesting on populations. We first used the mean MPM (Section 1.4) for each species, and simulated seed harvesting as a reduction in the values describing reproduction via seed in the sexual reproduction matrix ***F*** (see equation 1). Specifically, we created a modified MPM ***A’*** with reduced per-capita contributions of seed production in ***F***. To carry out our projections, we initiated the population vector ***n****_0_* as the stable stage distribution of the original MPM ***A***. This vector ***n****_0_* was obtained as the right-eigenvector of ***A*** following methods described by Caswell (2001). We then projected ***n****_0_* over 30 years using the modified MPM ***A’*** and the chain rule (Caswell 2001). We chose this period of time for our projections because it is long enough to observe even minor changes in the overall population size *N* that are not typically possible to quantify by short-term monitoring (Stott et al. 2011), while it is of sufficient length to fit within the active career of a land manager or conservation practitioner. We benchmarked the resulting population size *N*_30 harvest_ relative to the population size *N*_30 no harvest_ that would have been achieved in the absence of seed harvesting as in equation 2:

$$\begin{aligned} N_{30 relative}=\frac{N_{30 harvest}}{N_{30 no harvest}} \#eq.2 \end{aligned}$$

The relative population size *N*_30 relative_ thus ranges between 1 (when seed harvesting has no effect on population size; *N*_30 harvest_ = *N*_30 no-harvest_) to 0 (when the effect is so drastic it drives *N* to 0 within 30 years). For example, a value of *N*_30 relative_ = 0.1 means that the population size achieved with seed harvesting is 10% of the population size that would have been achieved without seed harvesting. The use of this metric as measure of seed harvesting impact allowed us to implement intra- and inter-specific comparisons, regardless of the variable population growth rates of each species’ population. When calculating the population sizes with and without harvest (*N*_30 harvest_ and *N*_30 no harvest_), we included only the active but not dormant (seed bank, dormant vegetative) life stages of the population vectors *N*_30_ because practitioners and scientists commonly evaluate population size based on counting active, standing individuals.

# Vulnerability to seed harvesting

We used mean MPMs to calculate species vulnerabilities to seed harvesting. For each species, we created 101 MPMs that describe the population dynamics when harvesting 0-100% of seed production, in 1% steps (Figure S1). As in section 2, we used the virtual MPMs to project population sizes over 30 years. We then fitted an exponential-decay model to quantify the effects of the varying proportion of harvested seed (p) on the relative population size in 30 years (*N*_30 relative_) as follows:

$$\begin{aligned} N_{30 relative}=e^{p\left( -b \right)} \#eq. 3 \end{aligned}$$

where *b* determines how steeply the relative population size (*N*_30 relative_) decreases with increasing proportion of harvest pressure, such that the larger *b*, the steeper this decrease is. We refer to this coefficient b as *vulnerability to seed harvesting* (Figure S2).


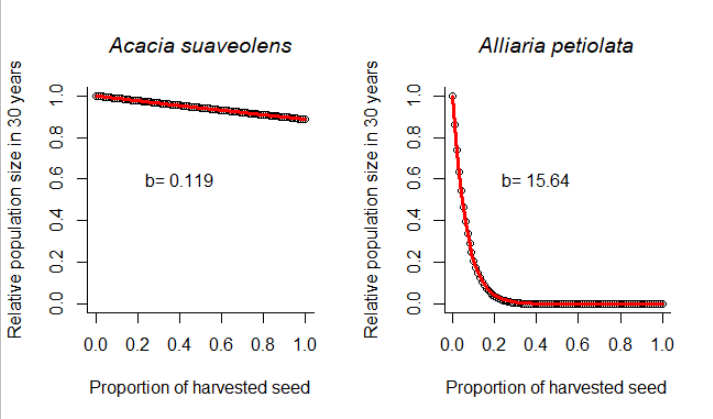


Figure S2. Vulnerability of population dynamics to seed harvesting (*b* in equation S3) in two of our 280 examined plant species. Note how the larger the value of *b*, the more vulnerable the given species is to seed harvesting. Black dots: simulated values; red line: fitted exponential-decay model as per equation 3.

# Testing current recommendations

Next, we used MPMs to simulate the impact of seed harvesting according to the current rules on the relative population size *N_30 relative_*. As far as we are aware of, explicit recommendations for the maximal proportion of seeds that can be harvested from natural populations so far exist only in three countries. In USA and Australia, this value is 20% and 10%, respectively, for common plant species when harvesting seeds for restoration projects (Plant Conservation Alliance 2015; Yenson et al. 2021). German rules are available for herbaceous plants: 2% for annual and 10% for perennial species when harvested every year (Prasse et al. 2010).

As the current recommendations are partly growth-form specific (Prasse et al. 2010), we examined the reduction in relative population size as a function of plant growth form: annuals, herbaceous perennials, epiphytes, lianas, palms, succulents, shrubs, and trees, as indicated in the COMPADRE metadata. We excluded growth forms represented by less than 5 species: epiphytes (n=4) and lianas (n=1), as well as plant species whose generation time disagreed with the metadata of the species, in particular annual species with generation times larger than two years (n=4). As the vulnerability to seed harvesting of individual species (Section 3) depended neither on a continent nor on the interaction between a continent and plant growth form (Table S2), we grouped species only by growth form and used the same set of species to test the recommendations from Australia, USA and Germany (Figure 1 in the main text).

Table S2: The effects of continent and plant growth form on vulnerability to seed harvesting. Results of a linear model with vulnerability to seed harvesting (log-transformed) as a response variable and plant growth form, continent, and their interaction as explanatory variables. We report results of a simple linear model because a generalized least square model with phylogenetic correction failed due to singular fit. The terms were fitted sequentially. Significant values are in bold. Adjusted R^2^=0.15

|  | df | resid. df | F | p |
| --- | --- | --- | --- | --- |
| Plant growth form | 5 | 243 | 10.37 | **<0.001** |
| Continent | 5 | 243 | 1.12 | 0.350 |
| Plant growth form × Continent | 13 | 243 | 0.99 | 0.466 |

# Life history traits

We used life history traits to explain species vulnerability to seed harvesting. A life history trait is a key feature that describes the life cycle of the organism (e.g. generation time, survival of seeds in the seed bank, clonal propagation). As our ultimate motivation was to facilitate the translation of our findings to land managers and practitioners, out of the wide range of life history traits that can be derived from MPMs (e.g Caswell 2001; Salguero-Gómez et al. 2016), we selected the traits that are readily available in trait databases or easy to estimate in the field (Table S3). All life history traits were calculated based on the matrix A of the mean MPM of each of our 280 species. For calculation of generation time, we selected the same method as in Salguero-Gómez et al. (2016), because this definition is broadly accepted and the generation time closely correlates with fast-slow gradient, which represents fast-growing, short-lived plant species at one end and slow-growing, long-lived species at the other.

Table S3: Formulation of the life history traits used to explain species vulnerability to seed harvesting in 280 vascular plant species. *λ* is the population growth rate, which corresponds to the dominant eigenvalue of the matrix *A*; l_x_ and m_x_ are stage-specific survival and fertility schedules, *C* is the submatrix describing clonal reproduction, m is the dimension of the matrix *C*, *w* is the stable stage distribution of the matrix *A*, with j column entries of the matrix population model.

| **Life history trait** | **Biological meaning** | **Formula** |
| --- | --- | --- |
| Generation time *T* | Number of years necessary for the individuals of a population to be fully replaced by new ones | $T=\frac{log(\int_{1}^{\infty} l_{x}m_{x}dx)}{log(\lambda)}$ |
| Degree of iteroparity *S* | Spread of reproduction throughout the lifespan of the individual as quantified by Demetrius’ entropy (S). High/low S values correspond to iteroparous/semelparous populations | $S= {-e}^{-log\lambda}l_{x}m_{x}log(e^{-log\lambda}l_{x}m_{x})$ |
| Age at sexual maturity *L_α_* | Number of years that it takes an average individual in the population to become sexually reproductive | L_α_ as described in Caswell 2001’s equation 5.41 (Caswell 2001) |
| Seed bank residence | Mean amount of time individuals are expected to stay in the seedbank stage | As described in Caswell 2001’s equation 5.36 (Caswell 2001) according to the fundamental matrix approach for the life cycle stage(s) that correspond to the seed bank stage(s) |
| Clonality Κ | Per-capita clonal contributions weighted by the stable stage distribution of the MPM | $K=\sum_{1}^{m} \bar{C}_{j}\bar{w}_{j}$ |

# The effect of life history traits on vulnerability to seed harvesting

We used linear models to determine which life history traits (generation time, degree of iteroparity, age at sexual maturity, seed bank residence, clonality) best explained species’ vulnerability to seed harvesting (Section 3). We also added plant growth form as an explanatory variable (as defined in the COMPADRE database, COMPADRE 2019)) to the model to test whether it explains any additional variability. Restricting the model to key life history traits allowed us to keep the full model and avoid model selection, which is known to produce exaggerated effect sizes and spurious effects (Leeb & Pötscher 2005). Species vulnerability to seed harvesting was log-transformed prior analysis to achieve normality. Other explanatory variables except plant growth type (factor) were log-transformed and standardised to adhere to the model assumptions of normally distributed errors.

To illustrate the importance of the life history traits for predicting the species vulnerability to seed harvesting (Figure 2 in the main text), we expressed the relative importance of each predictor in the model as the proportion of explained variability assigned to each predictor. As the explained variability can depend on the sequential order of the predictors in the model, we averaged the explained variability for each predictor across all possible ordering of the predictors using the R package *relaimp* (Grömping 2006). To visualize effect sizes of the effects of life history traits on species vulnerability to seed harvesting, as well as uncertainty of these effects, we used 95% credible intervals, a Bayesian analogue of confidence intervals. These were calculated from 10,000 simulations of the mean and variance of each estimate, using the *sim* function in the R package *arm* with non-informative prior (Korner-Nievergelt et al. 2015).

We also ran a model including the phylogenetic relationships among species to test the extent to which the explanatory power of life history traits on species’ vulnerability to seed harvesting is in fact driven by the phylogenetic inertia in plant life history traits (Blomberg et al. 2003). We used a phylogenetic generalized least square model to include the phylogeny of our species. We obtained the phylogeny from COMPADRE, following methods detailed elsewhere (Salguero-Gómez et al. 2016). With this model, we estimated Pagel’s *λ* (not to be confused with the population growth rate, also referred to as *λ* in the demographic literature, Caswell 2001), a measure of phylogenetic signal in the trait structure. Briefly, Pagel’s *λ*=0 indicates no effect of the phylogenetic structure in the dataset in explaining variation in a given trait, while Pagel’s *λ*=1 indicates that the phylogenetic structure perfectly predicts, i.e. is responsible for, the life history trait structure. Negative values suggest that closely related species have more different traits than would be expected by chance ((Blomberg et al. 2003). We found that the phylogenetic signal was overall weak and negative (Pagel’s *λ*=-0.1). Based on this result, we opted to present in this paper results from the linear model without phylogenetic correction (Table S4).

Table S4: The effects of life history traits on vulnerability to seed harvesting, with significant values (*P*<0.05) in bold. Adjusted R^2^ = 0.61.

|  | df | resid. df | F | p |
| --- | --- | --- | --- | --- |
| Generation time | 1 | 264 | 215.08 | **<0.001** |
| Degree of iteropartity | 1 | 264 | 6.34 | **0.012** |
| Age at sexual maturity | 1 | 264 | 12.30 | **<0.001** |
| Seed bank residence | 1 | 264 | 5.14 | **0.024** |
| Clonality | 1 | 264 | 10.17 | **0.002** |
| Plant growth type | 5 | 264 | 3.57 | **0.003** |

# Assessing limits of seed collection

We used the mean MPM per species to estimate what fraction of seed production one can collect from a natural population while only moderately affecting its dynamics. As a moderate effect we defined a reduction in population size *N* to not below 50% of the size that would have been achieved without seed harvesting during 30 years of a constant annual harvest intensity. While a reduction of population size by up to 50% over 30 years may seem relatively high, it corresponds to an annual decline of <2%. This threshold also allows for the persistence of the natural population under environmental stochasticity in >99% of species (see section 8.2).

For each species’ MPM, we simulated the effect of seed harvesting as a reduction of seed production transition by 0-100%, in 1% intervals. We used such reduced, virtual MPMs to simulate population dynamics across 30 years, and we recorded the final population size and expressed it as relative to population size that would be achieved without seed harvesting (see Section 2, note this calculation is the same as the first step of the calculation of vulnerability to seed harvesting, Section 3). Besides annual harvests, we also modelled the effect of harvesting seeds every 2, 5 or 10 years because reducing harvesting frequency up to once in 10 years is sometimes recommended to limit negative effects of seed harvesting on population dynamics (Menges et al. 2004). In this case, we modelled population dynamics with the original mean MPM while the reduced MPM was used every 2^nd^, 5^th^ or 10^th^ run. As the safe fraction for seed harvesting, we considered the largest proportion of seed that was possible to harvest without exceeding the 50 % reduction of the relative population size.

We related the safe fraction for seed harvesting to the generation time of plants – the most important predictor of species vulnerability to seed harvesting, which alone explained 52.3% of total variability in species vulnerability to seed harvesting. We used non-linear regression in *R* (*nsl*) to describe the sigmoid relationship between the safe fractions of seed harvesting and the generation time, and used function *PredFit* in package *investr* (Greenwell & Schubert Kabban 2014) to generate confidence intervals for the relationship (Figure 3 in the main text).

# Effect of environmental stochasticity

In a subset of our studied species, we simulated the effects of environmental stochasticity on population dynamics to understand how environmental stochasticity affects our prediction for seed harvesting based on mean MPMs. We used all species in our dataset represented by at least three individual MPMs (Section 1.4), resulting in 1,578 MPMs across 108 plant species. We simulated environmental stochasticity as projecting vector of stable stage distribution of the mean MPM by randomly drawn individual MPM in each step. To obtain a probability distribution of results under environmental stochasticity, we repeated this process 1,000 times. We expressed the results as *N*_30 relative_ (equation eq. 2). The effects of seed harvesting were simulated as above (Section 4), with the difference that in each of the 30 annual time-steps in each of the 1,000 simulation runs, we randomly drew an individual MPM from the set of individual MPMs available for a given species.

## The effect of seed harvesting on population size based on environmental stochasticity versus mean MPM

To understand how environmental stochasticity affected our results, we estimated the robustness of our results in stochastic environments. As an example, we used the effect of harvest of 20% of seed production, expressed as *N*_30 relative_, and simulated seed harvesting either using mean MPMs or stochastic simulation. We then compared the safe fraction for seed harvesting (*N*_30 relative_ > 0.5) based on the mean MPMs to the median of safe seed fraction based on the stochastic simulations.

The median of relative population sizes *N*_30 relative_ based on 1,000 permutations of stochastic simulations (y axis in Figure S3) closely correlated with the *N*_30 relative_ based on the mean MPMs. Interestingly, the relative population size *N*_30 relative_ based on stochastic simulation (blue points in Figure S3) was slightly higher than the *N*_30 relative_ based on mean MPMs (black line in Figure S3), especially in species that are more vulnerable to seed harvesting. Consequently, the safe fraction for seed harvesting based on the median of stochastic simulations was on average 0.017 higher that safe fraction based on the mean MPMs (Figure S3). This suggests that environmental stochasticity partly buffers the predicted decrease of population size caused by seed harvesting.


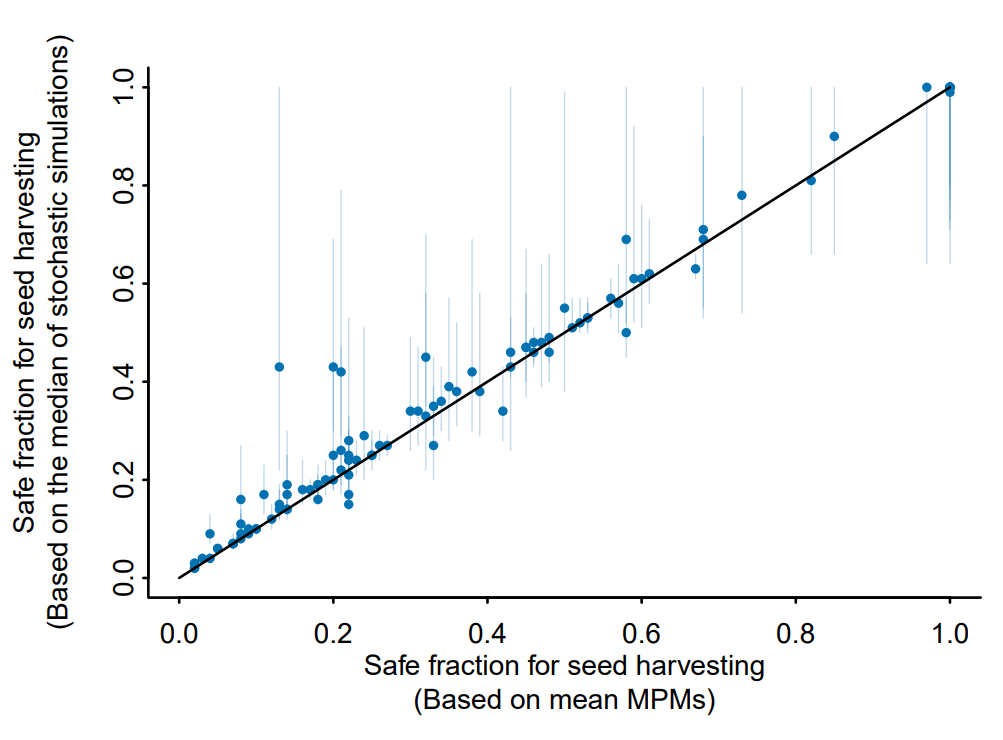


Figure S3: The safe fractions for seed harvesting based on *N_30 relative_* > 0.5 as calculated from the mean MPM (x-axis and 1:1 black line) versus the same safe fraction based on stochastic simulations (with 95% CI).

## Threshold for seed harvesting based on mean MPM versus extinction probability

In the models above, we set a threshold for seed harvesting so that the relative population size *N_30 relative_* decreased not below 50% of the population size that would be achieved without seed harvesting. In this section, we tested whether this threshold also prevented populations from extinctions. For each species, we computed what fraction of seeds could be sustainably harvested without causing extinction in at least 95% of stochastic simulations. We considered a population to go locally extinct when *N_30 relative_* < 0.01 (see Section 2 for definition of *N_30 relative_*). For each species, we compared the threshold based on the 95% probability of population survival with the threshold based on mean MPM and *N_30 relative_* > 0.5.

In the vast majority (>99%) of examined species, the threshold based on *N_30 relative_* > 0.5 (as calculated using mean MPMs, black line in the Figure S4) allowed for the collection of a lower proportion of seeds than the threshold based on 95% probability population survival when using stochastic simulations (individual points in Figure S4). This suggests that the rules based on *N_30 relative_* > 0.5 derived from the mean MPMs prevent populations from going locally extinct.


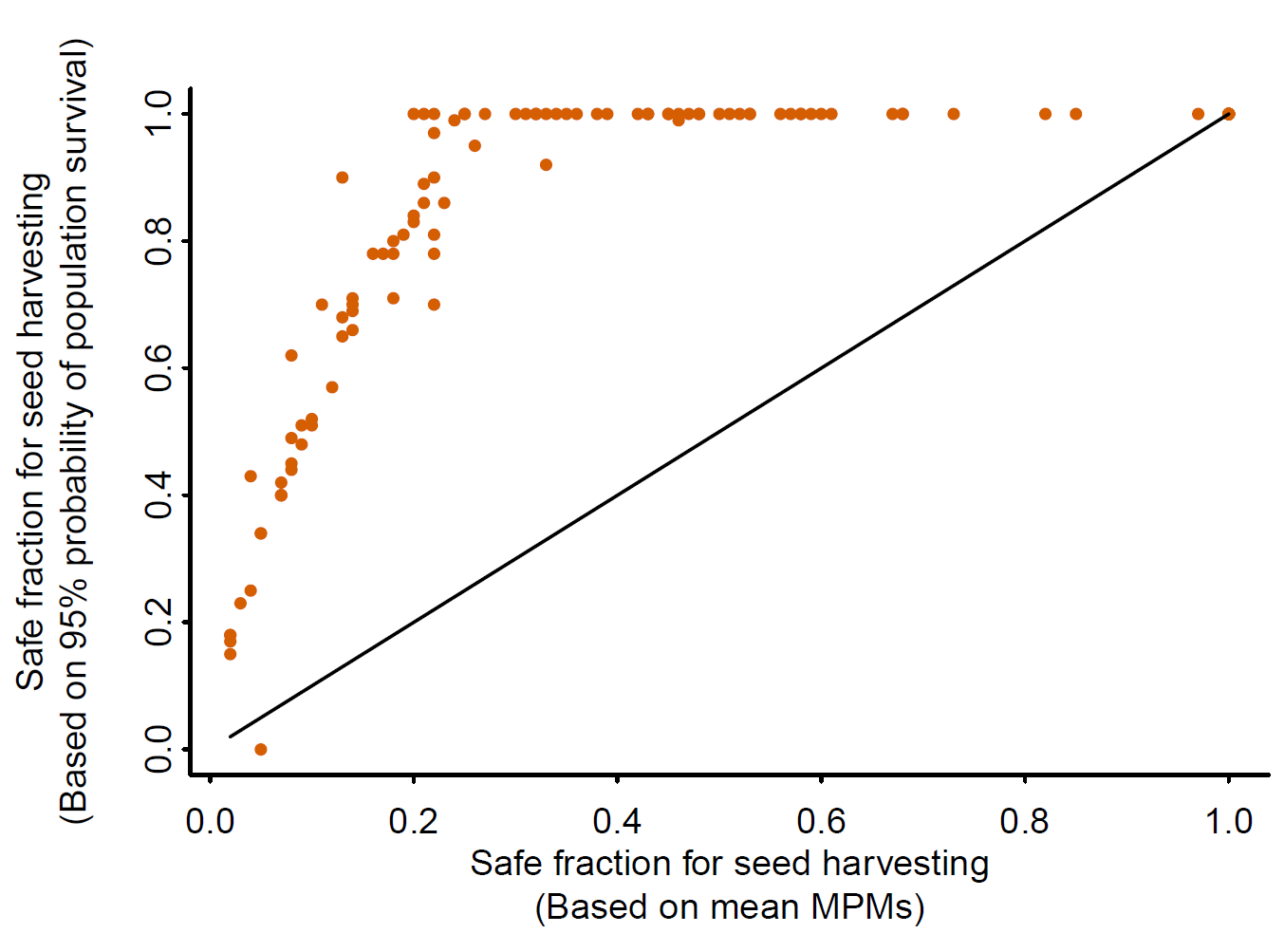


**Figure S4:** Comparison of the threshold for maximal seed harvest based on *N_30 relative_* > 0.5 as calculated from the mean MPM (x-axis and the 1:1 black line), with the maximal seed harvest that allows 95% probability of population survival of each considered species, as based on stochastic simulation.

**References**

Blomberg SP, Garland T, Ives AR. 2003. Testing for phylogenetic signal in comparative data: Behavioral traits are more labile. Evolution **57**:717–745.

Bucharová A, Brabec J, Münzbergová Z. 2012. Effect of land use and climate change on the future fate of populations of an endemic species in central Europe. Biological Conservation **145**:39–47.

Caswell H. 2001. Matrix population models: construction, analysis, and interpretation. Sinauer Associates, Sunderland, Massachusetts.

COMPADRE Plant Matrix Database (2019). Available from: https://www.compadre-db.org [25.8.2019, Version 5.0.0]. 2019, August 25.

de Kroon H, Plaisier A, van Groenendael J, Caswell H. 1986. Elasticity: The Relative Contribution of Demographic Parameters to Population Growth Rate. Ecology **67**:1427–1431.

de Kroon H, van Groenendael J, Ehrlen J. 2000. Elasticities: A review of methods and model limitations. Ecology **81**:607–618.

Greenwell B, Schubert Kabban Ch. 2014. investr: An R Package for Inverse Estimation. The R Journal **6**:90–100.

Grömping U. 2006. Relative Importance for Linear Regression in *R* : The Package **relaimpo**. Journal of Statistical Software **17**:1–27.

Kattge J et al. 2011. TRY - a global database of plant traits. Global Change Biology **17**:2905–2935.

Korner-Nievergelt F, Roth T, von Felten S, Guelat J, Almasi B, Korner-Nievergelt P. 2015. Bayesian data analysis in ecology using linear models with R, BUGS, and Stan. Elsevier.

Leeb H, Pötscher BM. 2005. Model selection and inference: Facts and fiction. Econometric Theory **21**:21–59..

Menges ES. 1990. Population Viability Analysis for an Endangered Plant. Conservation Biology **4**:52–62.

Menges ES, Guerrant EO, Hamze S. 2004. Effects of seed collection on the extinction risk of perennial plants. Pages 305–324 in Guerrant EO, Havens K, Maunde M, editors. Ex situ plant conservation: supporting species survival in the wild. Island Press, Washington.

Morris WF, Doak DF. 2002. Quantitative conservation biology : theory and practice of population viability analysis. Sinauer Associates.

Nguyen V, Buckley YM, Salguero-Gómez R, Wardle GM. 2019. Consequences of neglecting cryptic life stages from demographic models. Ecological Modelling **408**:108723. Elsevier.

Plant Conservation Alliance. 2015. National seed strategy for rehabilitation and restoration. Available from https://www.fs.fed.us/wildflowers/Native_Plant_Materials/documents/SeedStrategy081215.pdf.

Prasse R, Kunzmann D, Schröder R. 2010. Development and practical implementation of minimal requirements for the verification of origin of native seeds of herbaceous plants (in German). In cooperation with Verband Deutscher Wildsamen- und Wildpflanzenproduzenten. DBU, reference no. 23931, 166p.

R Development Core Team. 2015. R: A language and environment for statistical computing. Vienna.

Salguero-Gómez R, Jones OR, Jongejans E, Blomberg SP, Hodgson DJ, Mbeau-Ache C, Zuidema PA, de Kroon H, Buckley YM. 2016. Fast-slow continuum and reproductive strategies structure plant life-history variation worldwide. Proceedings of the National Academy of Sciences of the United States of America **113**:230–5.

Stott I, Townley S, Hodgson DJ. 2011. A framework for studying transient dynamics of population projection matrix models. Ecology Letters **14**: 959-970

Yenson MAJ et al. 2021. Plant Germplasm Conservation in Australia: strategies and guidelines for developing, managing and utilising ex situ collections. Third edition. Australian Network for Plant Conservation, Canberra.
